# Genome-wide identification and functional validation of sterol C-22 desaturases and C-24 methyltransferases in *Asparagus officinalis* and *Asparagus taliensis*

**DOI:** 10.3389/fpls.2025.1690526

**Published:** 2025-11-18

**Authors:** Sylvia E. Brown, Yunbin Li, Chun Lin, Zhengjie Liu, Zichao Mao

**Affiliations:** 1 College of Agronomy and Biotechnology, Yunnan Agricultural University (YNAU), Kunming, China; 2 Institute of Improvement and Utilization of Characteristic Resource Plants, Yunnan Agricultural University (YNAU), Kunming, China; 3 The Laboratory for Crop Production and Intelligent Agriculture of Yunnan Province, Kunming, China

**Keywords:** sterol biosynthesis, sterol C-22 desaturase, sterol C-24 methyltransferase, *Asparagus officinalis*, *Asparagus taliensis*, functional genomics, expression profiling, stress resilience

## Abstract

**Introduction:**

Steroids are essential components of plant membranes and serve as precursors of brassinosteroids (BRs) and Steroidal saponins (SSs), which regulate growth, development and stress adaptation. Sterol-modifying enzymes, including C-22 desaturases (C22SDs) and C-24 methyltransferases (C24SMTs), act as key branch-point regulators of side-chain remodeling, yet their molecular roles in *Asparagus* remain poorly characterized.

**Methods:**

Functional genomics analysis of C22SD and C24SMT families was conducted in *Asparagus officinalis* and *A. taliensis*, integrating genome-wide identification, phylogenetic reconstruction, gene structure, conserved motif and cis-element analyses. Transcriptomics-based expression profiling revealed tissue-specific expression patterns, supporting functional divergence among gene family members. Structural modeling and molecular docking highlighted conservation of catalytic residues and predicted substrate interactions.

**Results:**

To overcome transformation barriers in *Asparagus*, functional validation was performed in *Neurospora crassa*, where targeted disruption of *erg5*, *erg6* or both impaired ergosterol biosynthesis, growth and membrane fluidity, while complementation with selected *Asparagus* genes restored these traits. The results catalog C22SD/C24SMT families and show heterologous complementation of Δerg5, Δerg6 and Δerg5/Δerg6 mutants in *N. crassa*, indicating catalytic competence in eukaryotic sterol pathway, in-planta roles remain to be established.

**Discussion:**

By characterizing sterol side-chain remodeling enzymes, this study establishes a framework for understanding the potential roles of these enzymes in membrane stability, hormone biosynthesis and defense metabolite production, with implications for stress resilience and metabolic engineering. These findings highlight sterol remodeling as a potential target for developing stress-resilient crops.

## 1 Introduction

Steroids are indispensable components of eukaryotic membranes, where they regulate fluidity, permeability and protein function. In plants, sterols also serve as precursors to a variety of bioactive metabolites, most notably brassinosteroids (BRs) and steroidal saponins (SSs) (Cheng et al., 2023; Li et al., 2023; Wang et al., 2022; Williams and Gong, 2007). The emergence of oxygen-dependent sterol biosynthesis is thought to have been linked to the rise in atmospheric oxygen, shaping eukaryotic evolution (Hoshino and Gaucher, 2021; Shirke et al., 2025). Unlike prokaryotic hopanoids, which are synthesized in a single oxygen-independent step from squalene (Pan et al., 2015), plant sterols are formed through complex oxygen-dependent pathways that produce 24-methylsterols, 24-ethylsterols and cholesterol (CHOL), thereby connecting membrane structure with hormone signaling and specialized metabolism (Sonawane et al., 2016).

The biosynthesis of BRs and SSs shares common origins from 2,3-oxidosqualene and involves several branch-point enzymes such as cycloartenol synthase, sterol methyltransferases (SMTs) and cytochrome P450 monooxygenase (Christ et al., 2019; Sonawane et al., 2016; Wei and Li, 2016). Among these, sterol C-22 desaturases (C22SDs) and sterol C-24 methyltransferases (C24SMTs) are key regulators of side-chain remodeling. C22SDs introduce a double bond at the C-22(23) position, diversifying BR precursors, while C24SMTs catalyze methylation at the C-24 position to generate C-24 methyl and ethyl sterols via SMT1 and SMT2 isoforms (Guan et al., 2017; Neelakandan et al., 2010; Ohta and Mizutani, 2013; Shirke et al., 2025). Their coordinated activity influences the metabolic flux between BR and SS pathways, ultimately affecting growth, development and defense.

Importantly, sterol composition is closely linked to plant stress resilience. Membrane sterols stabilize lipid bilayers, buffering plants against environmental fluctuations by maintaining proper fluidity under heat, cold, drought or salinity (Aboobucker and Suza, 2019; Shirke et al., 2025). BRs act as growth-promoting hormones that also enhance tolerance to multiple abiotic stresses (Shirke et al., 2025; Wang et al., 2022), while SSs function as antimicrobial and deterrent compounds that contribute to biotic stress defense (Shakeel et al., 2025). Thus, enzymes that control sterol metabolism occupy a central position in shaping plant adaptation strategies under diverse stress conditions.

The genus *Asparagus* (Asparagaceae) includes both the globally cultivated vegetable *A. officinalis* and the medicinal plant *A. taliensis*. These species accumulate steroidal metabolites such as brassinosteroids and saponins, notably involved in development and phytochemical defense (Cheng et al., 2023). Their mitogenomic diversity also reflects ecological adaptation and domestication dynamics (Wu et al., 2024). Although C22SDs and C24SMTs have been functionally characterized in several model crop species, their roles in *Asparagus* remain uncharacterized. In *Arabidopsis thaliana*, CYP710A1/2 catalyze the Δ22-desaturation of sitosterol and campesterol, producing stigmasterol and brassicasterol, which contribute to developmental regulation and abiotic stress responses (Morikawa et al., 2006). In tomato, CYP710A11 (LeSD1) expression is associated with ripening specific increases in stigmasterol content, linking sterol desaturation with fruit developmental processes (Whitaker and Gapper, 2008). In soybean, modulation of SMT activity alters membrane sterol composition (Neelakandan et al., 2010). Moreover, pathogen infection has been shown to shift the β-sitosterol/stigmasterol ratio, such as in soybean roots infected by *Meloidogyne incognita* (A. Cabianca et al., 2021), suggesting a potential link between sterol metabolism and biotic stress responses. Despite this progress in other systems, no genome-wide identification or catalytic validation of these enzymes has been reported in *Asparagus*. This gap is significant given that *A. officinalis* is a globally important vegetable crop, while *A. taliensis* and related species are notable for their rich accumulation of SSs with documented pharmacological value (Cheng et al., 2023; Wu et al., 2024). By addressing this gap, our study provides the first molecular and preliminary catalytic evidence of sterol-modifying enzymes in *Asparagus*, situating sterol remodeling within the unique dual agricultural and medicinal significance of this genus. Recent work emphasizes how BR signaling integrates with other hormones during development and stress adaptation, underscoring the translational potential of sterol remodeling (Guo et al., 2024).

Due to the complexities of transformation in *A. officinalis* (Chen et al., 2019) and the absence of an established system in *A. taliensis*, *Neurospora crassa* (*N. crassa*) was employed as a heterologous host to validate enzymatic activity (Borkovich et al., 2004; Davis and Perkins, 2002). Heterologous assays in *N. crassa* provide a tractable readout of sterol-pathway activity and are interpreted solely as evidence of catalytic competence, not as evidence for plant stress biology, pending in-planta validation.

This study integrates comparative genomics and functional validation of C22SD and C24SMT enzymes from two *Asparagus* species, with the following specific aims: (I) identify and select representative C22SD and C24SMT genes for cloning and construct design. (II) Perform structural characterization through homology modeling and molecular docking to evaluate substrate interactions with ergosta-5,7,24(28)-trienol and zymosterol. (III) Assess catalytic function by disrupting *erg5* and *erg6* in *N. crassa* as single and double mutants, followed by complementation with selected *Asparagus* genes and (IV) Quantify phenotypic rescue by measuring growth rates, membrane fluidity and ergosterol (ERG) levels, using gas chromatography-mass spectrometry (GC-MS).

Collectively, these efforts provide the first comprehensive molecular and preliminary catalytic evidence of sterol-modifying enzymes (C22SD and C24SMT) in *Asparagus*, establishing a framework for understanding their potential roles in BR and SS metabolism and laying a foundation for metabolic engineering and crop improvement.

## 2 Materials and methods

### 2.1 Plant materials and RNA-seq resources

Roots (Rs), stems (Ss) and flowering (Fs) of *A. officinalis* and *A. taliensis* were collected from field-grown plants at Yunnan Agricultural University (Kunming, Yunnan, China). For each species and tissue, three biological replicates (n = 3) were harvested, immediately flash-frozen in liquid nitrogen and stored at -80°C until use. Transcript-level expressions of candidate C22SD and C24SMT genes was quantified from RNA-seq datasets generated in previous studies (Cheng et al., 2023; Wu et al., 2024) and deposited in the China National Center for Bioinformation (CNCB) under BioProjects; PRJCA011702 (*A. officinalis*) and PRJCA011431 (*A. taliensis*).

### 2.2 *In silico* identification and phylogenetic analysis of C22SD and C24SMT families

Candidate *C22SD* and *C24SMT* genes were identified from the genomes of *A. officinalis* (Phytozome; Aofficinalis_V1.1) and *A. taliensis* (CNCB; Genome Warehouse, accession: GWHBKKQ00000000). Hidden Markov profiles for cytochrome P450 (PF00067) and Sterol_MT_C (PF08498) were retrieved from InterPro (Blum et al., 2024) and queried using HMMER v3.4 (Finn et al., 2011). Candidates were further screened by BLASTp (Blast+ v2.11.0) against representative viridiplantae proteomes from National Center for Biotechnology Information (NCBI) (species listed in **Supplementary Table S1**). Sequences were retained using E-value ≤ 1e-5, identity ≥ 40% and alignment coverage ≥ 50%. Domain architectures were verified with InterPro. Ortholog sets (**Supplementary Table S1**) were aligned with MUSCLE (Edgar, 2004), and phylogenies were inferred in MEGA 11 (Tamura et al., 2021) using the neighbor-joining method with 1000 bootstrap replicates and pairwise deletion of sites containing gaps or missing data. Neighbor-joining trees are used only for descriptive placement of *Asparagus* sequences among characterized sterol enzymes. Protein length, theoretical molecular weight and isoelectric point were computed in TBtools (Chen et al., 2023), while chromosomal coordinates were parsed from general feature format version 3 (GFF3) annotations. Subcellular localization was predicted with CELLO (Yu et al., 2006). All physiochemical parameters are summarized in **Supplementary Table S2**.

### 2.3 Conserved motif, gene structure and cis-regulatory element analysis

Conserved motifs in C22SD and C24SMT proteins were identified using multiple expectation maximization for motif elicitation (MEME) v5.0.5 (maximum motifs: 10, width: 6-50, occurrence model: ZOOPS, (Zero or One Occur Per Seq.). Gene structure (exon/intron organization) was visualized from GFF3 and CDS files in TBtools (Gene structure view-Advanced tool), using the longest isoform per gene. Promoters were defined as the 2000 bp upstream of the annotated ATG. Cis-regulatory elements were annotated with PlantCARE (Lescot et al., 2002) and grouped into functional categories (e.g., hormone, stress and light responsiveness). Heatmaps of per gene counts were generated to summarize regulatory potential.

### 2.4 Expression profiling and co-expression network analysis

RNA reads (Illumina NovaSeq 6000) were quality-checked with FastQC (de Sena Brandine and Smith, 2019), filtered with Fastp (https://github.com/OpenGene/fastp), and aligned to the respective reference genomes using Hisat2 (Kim et al., 2015). Gene-level quantification was performed with FeatureCounts to generate read count and transcripts per kilobase of exon model per million reads (TPM) expression matrices transcript abundance of C22SDs and C24SMTs was compared across Rs, Fs and Ss and visualized using TBtools (Chen et al., 2023).

Weighted gene co-expression network analysis (WGCNA) (Langfelder and Horvath, 2008) was used to infer candidate regulatory interactions based on three criteria: (i) co-localization in expression modules, (ii) strong correlation (absolute R >0.7, p-value < 0.05), and (iii) the presence of predicted cis-regulatory motifs for correlated TFs in promoter regions.

### 2.5 Protein structure prediction and their molecular docking

Three-dimensional (3D) structures of candidate C22SD and C24SMT proteins were predicted with AlphaFold 3 (Abramson et al., 2024). Docking was performed using CB-Dock2 (Liu et al., 2022) with ergosta-5,7,24(28)-trienol as the ligand for C22SDs and zymosterol for C24SMTs. Binding affinities (kcal/mol) were recorded, and the docked complexes were visualized in PyMOL (Bramucci et al., 2012). Structural similarity to fungal homologs (*N. crassa* erg5/erg6) was quantified by root mean square deviations (RMSDs) and PyMOL MatchAlign scores.

### 2.6 Gene isolation, cloning and vector construction

Total RNA was extracted using the RNA Easy Fast Plant Tissue Kit (Tiangen, China) and reverse transcribed with FastKing gDNA Dispelling RT SuperMix (Tiangen, China). Full-length coding sequences (CDS) of selected C22SD and C24SMT genes were amplified with gene-specific primers (**Supplementary Table S3**), cloned into T-vectors (Yeasen Biotech, China) and sequenced. Verified inserts were re-amplified with restriction sites *BssH*II at 5’ ends and both *BamH*I and *Xma*I sites at the 3’ ends. After double digestion with *BssH*II and *Xma*I, the fragments were inserted into the corresponding sites of the pcfp_Myc_Bar and pcfp_Myc_Hph expression vectors, under the control of the *cfp* prompter, derived from the pyruvate decarboxylase gene, known for its constructive activity (Temporini et al., 2004). This resulted in intermediate vectors (listed in **Supplementary Table S4**). Subsequently, the glyceraldehyde-3-phosphate dehydrogenase terminator (T_gpdh) from *N. crassa* (Shinohara et al., 1998) was amplified, double digested with *BamH*I and *Xma*I and ligated into the respective sites of the intermediate vectors. This yielded the final expression vectors: pAofC22SD, pAtaC22SD, pAofC24SMT and pAtaC24SMT (**Supplementary Table S4**; see **Supplementary Figure 10** for a step-by-step schematic).

### 2.7 Targeted erg5/erg6 disruption and complementation in *N. crassa*

Targeted replacement of erg5 and erg6 were carried out using PCR-based gene replacement methods (Collopy et al., 2010; Colot et al., 2006) with minor modifications. Disruption cassettes comprised ~1.2 kb gene-specific 5’ and 3’ flanking regions fused to selectable markers; *hph* for *erg5* and *bar* for *erg6*. The selectable markers were driven by the *Aspergillus nidulans trpC* promoter and terminated by *T_gpdh* from *N. crassa* (Shinohara et al., 1998). The 5’ and 3’ flanks for each locus were amplified by PCR from genomic DNA (gDNA) of strains Ku70 (Mat a) and 301-6 (Mat A) using gene-specific primers (**Supplementary Table S3**). Full-length disruption cassettes were assembled by overlap-extension PCR (Cha-Aim et al., 2012) in the order 5’ flank - selective marker (trpC::hph::T_gpdh or trpC::bar::T_gpdh) - 3’ flank, cloned into T-vector for sequence verification and purified for transformation. Purified cassettes (~50 ng per transformation) were introduced into *N. crassa* ku70 and 301–6 by electroporation (Li et al., 2017). Spore suspensions (~2.5x10^9^ spores/mL) were mixed with the purified cassettes and pulses in 1 mm gap cuvettes (1.5 kV, single pulse). Immediately, post-pulse, 1 mL 1M sorbitol was added, cells were recovered at 30°C for 1.5 - 2h and plated on vogel’s minimal medium agar (Metzenberg, 2003), supplemented with the appropriate antibiotic. Colonies that appeared after 5–7 days were streak-purified and transferred to vogel’s minimal agar slants containing the same antibiotic(s) for propagation. The gDNA was extracted and successful gene disruption was verified by PCR amplification across flanking and internal regions using specific primers (**Supplementary Table S3**).

To generate the Δerg5/erg6 double knockout strain, verified single disrupted mutants Δerg5 (Mat A) were crossed on standard *N. crassa* crossing medium. After incubation at 28°C for 14 days, perithecia were collected and ascospores were heat-shocked at 50°C for 10 min to induce germination. Ascospores were plated on vogel’s minimal media containing both *hph* and *bar* for double selection. Resulting colonies were screened by PCR to verify disruption of both erg5 and erg6.

For functional complementation, expression constructs carrying *A. officinalis* and *A. taliensis* C22SD and C24SMT genes were introduced into the corresponding mutant backgrounds: C22SD vectors (pAofC22SD and pAtaC22SD) into Δerg5, C24SMT vector (pAofC24SMT and pAtaC24SMT) into Δerg6 and combinations of both into Δerg5/erg6. Vector-only transformants were included as negative controls to exclude potential effects of the vector backbone. Following electroporation, transformants were selected on the same antibiotic regimens (*hph* and *bar*) at 30°C for 3–5 days and subcultured gDNAs were used to confirm transgenes integration by PCR with gene-specific primers (**Supplementary Table S3**). Mating types was verified by PCR with mating type diagnostic primers (Huang et al., 2018).

Total RNA from *N. crassa* strains was extracted using the HiPure Total RNA Kit (Magen Biotech, China), treated with DNase I, and reverse-transcribed with FastKing gDNA Dispelling RT SuperMix (Tiangen Biotech, Beijing, China) according to the manufacturer’s protocols. Reactions were run on an ABI 7500 Fast Real-Time PCR system using SuperReal PreMix Plus (SYBR Green) (Tiangen Biotech, Beijing, China). Thermal profile: 95°C for 30s; 40 cycles of 95°C for 10s and 60°C for 30s; melt analysis: 95°C for 15s, 60°Cfor 60s,95°C for 15s. Each sample included three biological replicates, with each replicate run in triplicate. Two assays were performed: (i) endogenous assays for *N. crassa* erg5 or erg6 using gene-specific primers and (ii) transgene assays for *Asparagus* C22SD or C24SMT using transgene-specific primers. Primer sequences are listed in **Supplementary Table S3**. Actin served as the internal reference gene. Relative expression was calculated within each assay using 2^-ΔΔCT^ method (Schmittgen and Livak, 2008).

### 2.8 Sterol extraction and GC-MS quantification of ergosterol

Mycelia from 3 days liquid cultures were harvested, freeze-dried and homogenized in chloroform:methanol (2:1, v/v) (Salazar Alekseyeva et al., 2021). After phase separation, the organic layer was evaporated to dryness using a rotary evaporator, and dried sterol residues were derivatized with N,O-bis(trimethylsilyl)trifluoroacetamide (BSTFA, with 1% Pyridine) at 70°C for 1h. Derivatives were analyzed using a gas chromatography-mass spectrometry (GC-MS) system consisting of an AGILENT 7890 gas chromatograph coupled to a 5975 type mass selective detector, using a DB-5MS capillary column (30 m x 0.25 mm x 0.25 μm). Helium was used as the carrier gas at a constant flow rate of 1.2 mL/min. the oven program was set as follows: initial temperature 100°C, ramped at 20°C/min to 280°C, then held for 10 min. the inlet temperature was maintained at 280°C and the mass spectrometry (MS) transfer line was set at 290°C. MS acquisition was performed in electron impact (EI) mode, scanning from m/z 50 to 600.

ERG was identified by retention time and EI spectra relative to an authentic ergosterol standard (CAS: 57-87-4, ACMEC Biochemicals, Shanghai, China) and quantified against a 5-point calibration curve, producing the linear regression Y = 2x10^8^ X – 2x10^7^ (X: relative intensity, Y: µg/ml ERG, R² = 0.999). this method enabled comparative analysis of ERG biosynthesis across wild-type, disrupted and complementary strains, reflecting the influence of C22SD and C24SMT gene expression on sterol biosynthesis.

### 2.9 Phenotypic characterization

#### 2.9.1 Growth rate comparison

Growth rates were evaluated by culturing fungal strains in 500 mL Erlenmeyer flasks containing 100 mL of vogel’s minimal medium, supplemented with glucose and vogel’s salts. Spore suspensions were prepared from wild-type and transformed strains grown on vogel’s minimal agar slants and adjusted to a final concentration of~1x10^6^ spores/mL. Each flask was inoculated with 1 mL of the spore suspension and incubated at 30°C with shaking at 180 rpm. For each strain, three biological replicates were maintained. Fungal biomass was harvested at 6 time points (2, 4, 6, 8, 10 and 12 days post inoculation). At each time point, mycelia were filtered, rinsed with distilled water and dried at 60°C to a constant weight. Dry biomass (g/L) was recorded and growth curves were constructed by plotting dry weight against incubation time in days. The experimental phase growth rates were calculated during the exponential growth phase using the formula:


$$\text{Exponential phase growth rate}\;(\mu )=\frac{\ln(W_{2})-\ln(W_{1})}{t_{2}-t_{1}}$$

Where: *W_1_*& *W_2_* are the biomass concentration (dry weights, g/L) at *t_1_* and *t_2_* respectively, and *t_1_* and *t_2_* are the time points (in days) at which the biomass was measured.

#### 2.9.2 Hyphae morphology comparison

To assess hyphal morphology, wild-type, disrupted and complemented strains were inoculated onto vogel’s minimal agar medium. The cultures were incubated at 30°C under a 14 h light/10 h dark cycle for 7 days. After incubation, the cultures were visually examined for differences in hyphal coloration and density.

#### 2.9.3 Membrane fluidity assay

Spore suspensions (~1x10^6^ spores/mL) were incubated with 1 µM 1,6-diphenyl-1,3,5-hexatriene (DPH) for 30 min in the dark at room temperature. After staining, spores were washed three times to remove unbound dye, enhancing fluorescence measurement accuracy. Fluorescence intensity was measured using a SpectraMax ID3 plate reader with excitation and emission wavelengths set to 360 nm and 430 nm respectively (Santos et al., 2017). Measurements were performed in 96-well plates (height: 14.6 mm) with a 5s orbital shake prior to reading. Readings were taken from the top of the wells at a height of 1.00 mm. Temperature was maintained at 30°C (mean temperature during measurements was 30.5°C). Membrane fluidity was quantified by calculating the fluidity index using the formula:


$$\text{Fluidity Index }(\text{FI})=\frac{\text{Fluorescence intensity }(\text{Strain})}{\text{Fluorescence intensity }(\text{wildtype})}$$

### 2.10 Statistical analysis

All experiments were conducted with at least three independent biological replicates (n = 3). Data are presented as means, with error bars in figures representing the standard error of the mean (SEM). Statistical significance was assessed using one-way ANOVA followed by Tukey’s *post hoc* test for multiple comparisons. A threshold of p < 0.05 was considered statistically significant. All statistical analysis and visualization were performed in GraphPad Prism v9.0.

## 3 Results

### 3.1 *In silico* identification and phylogenetic analysis

**Figure 1:**
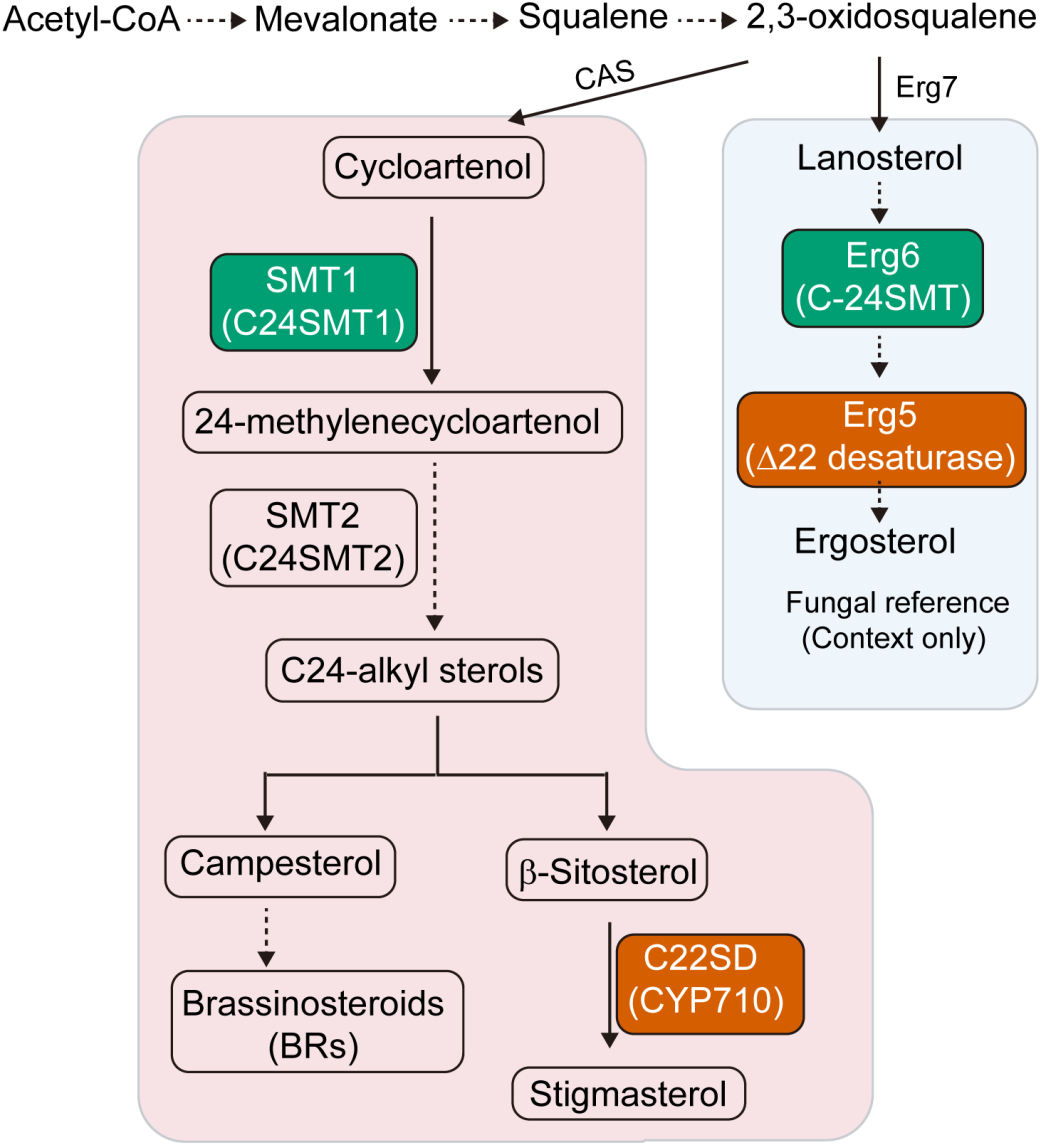
Sterol side-chain remodeling pathway in plants and fungi. Schematic representation of sterol biosynthesis showing focal enzymes characterized in this study: sterol C-24 methyltransferase (SMT1/C24SMT) and sterol C-22 desaturase (C22SD/CYP710). The plant pathway (left) proceeds from acetyl-CoA via the mevalonate pathway to 2,3-oxidosqualene, cyclized by cycloartenol synthase (CAS) to cycloartenol. Side-chain modifications by SMT1 (C24SMT1) and SMT2 (C24SMT2) produce C24-alkyl sterols including β-sitosterol, which is converted by C22SD into stigmasterol. Brassinosteroids (BRs) are derived from campesterol. The fungal reference pathway (right, shown in blue) illustrates the lanosterol-derived route to ergosterol, highlighting Erg6 (C-24SMT) and Erg5 (Δ22-desaturase) for homology context. Solid arrows represent single enzymatic steps and dashed arrows indicate multi-step conversions.

**Figure 2:**
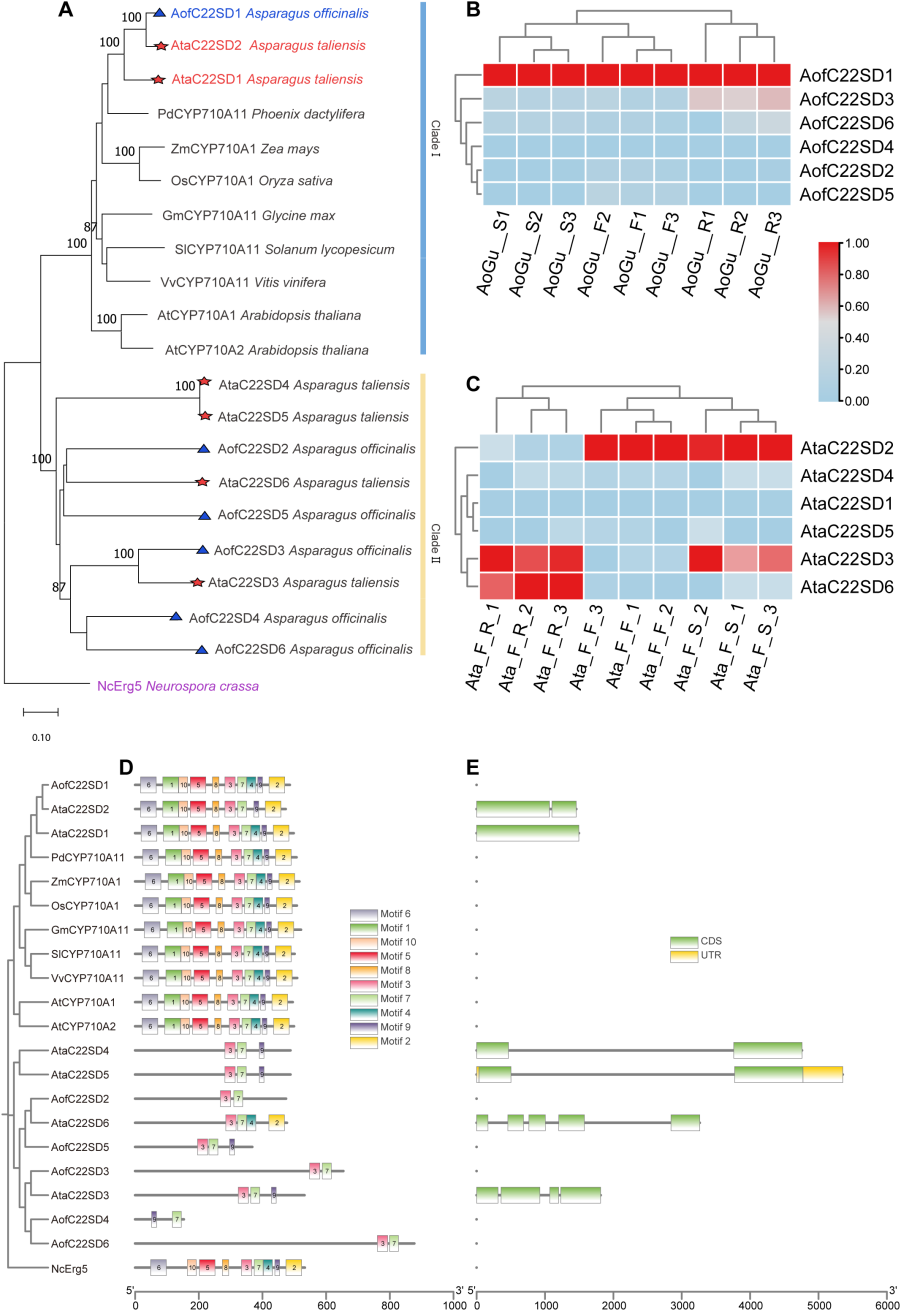
C22SD family in Asparagus with phylogeny, expression, motif and gene structure context. **(A)** Neighbor-joining phylogeny of C22SD candidates from A. officinalis (blue) and A. taliensis (red) alongside reference plant sequences, rooted with Neurospora crassa erg5 (NcErg5, purple) as outgroup. Bootstrap support values ≥ 80% (1000 replicates) are indicated at nodes. Clade I (blue) and clade II (yellow) are indicated by right hand bars. The scale bar represents 0.10 substitutions per site; **(B, C)** Tissue-specific expression profiles (TPM, log2-scaled, normalized 0–1 per gene) across roots (Rs), stems (Ss) and flowering twigs (Fs) in A. officinalis **(B)** and A. taliensis **(C)**. Heatmaps display triplicate samples: A. officinalis (green cultivar) roots (AoGu_R1-3), stems (AoGu_S1-3), flowering twigs (AoGu_F1-3); A. taliensis female roots (Ata_F_R1-3), female stems (Ata_F_S1-3), female flowering twigs (Ata_F_1-3). **(D)** Conserved motifs architecture (MEME) showing retention and order of catalytic motifs; AofC22SD1 and AtaC22SD2 retain the full motif set, supporting catalytic conservation across orthologs; **(E)** Exon-intron organization (longest isoforms per gene). Together, phylogenetic placement, motif integrity, and tissue expression nominate AofC22SD1 and AtaC22SD2 as lead candidates for experimental validation.

**Figure 3:**
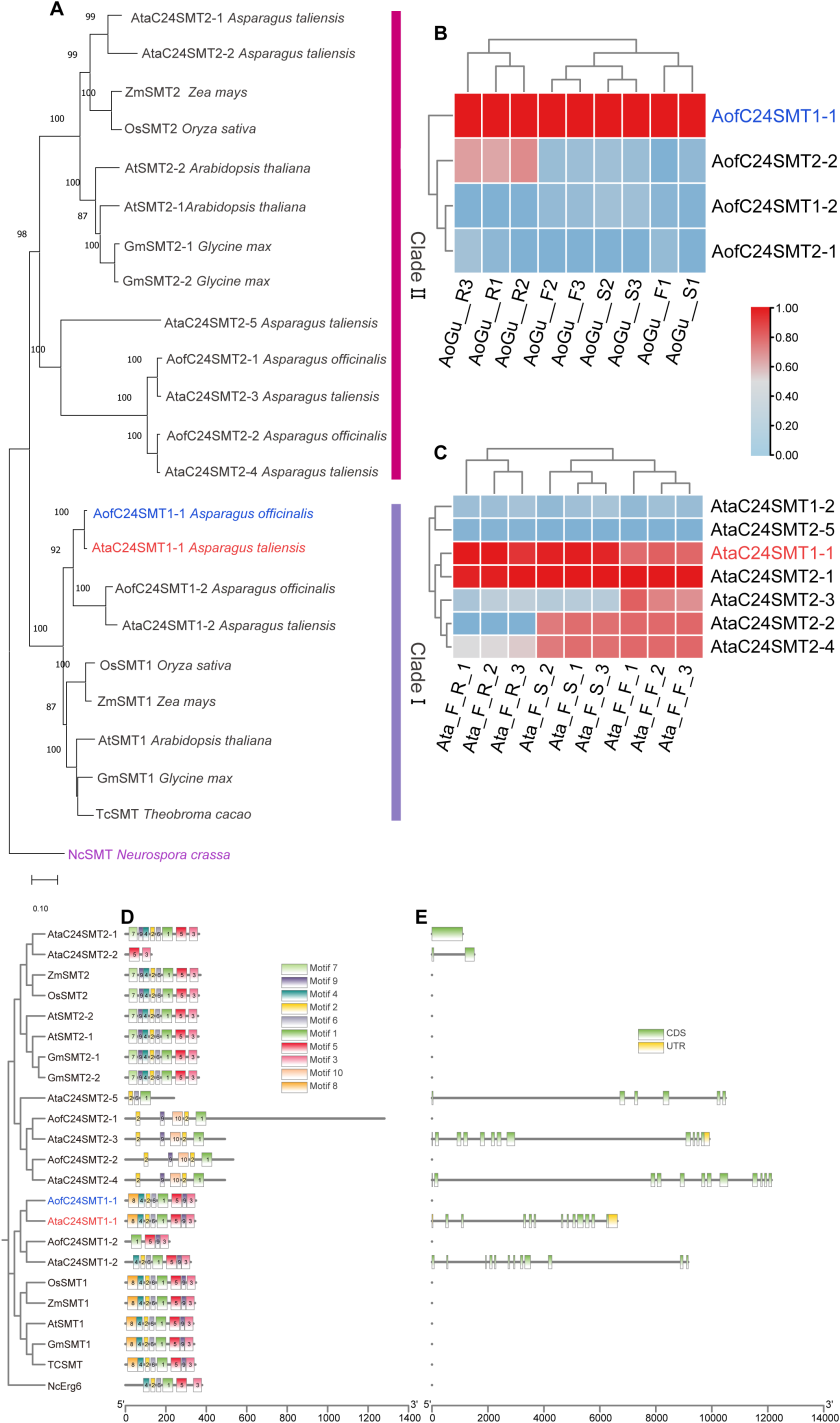
C24SMT family in Asparagus with phylogeny, expression, motif and gene structure context. **(A)** Neighbor-joining phylogeny of C24SMT candidates from A. officinalis (blue) and A. taliensis (red) alongside reference plant sequences, rooted with Neurospora crassa erg6 (NcErg6, purple) as outgroup. Bootstrap support values ≥ 80% (1000 replicates) are indicated at nodes. Clade I (red) and clade II (purple) are indicated by right hand bars. The scale bar represents 0.10 substitutions per site; **(B, C)** Tissue-specific expression profiles (TPM, log2-scaled, normalized 0–1 per gene) across roots (Rs), stems (Ss) and flowering twigs (Fs) in A. officinalis **(B)** and A. taliensis **(C)**. Heatmaps display triplicate samples: A. officinalis (green cultivar) roots (AoGu_R1-3), stems (AoGu_S1-3), flowering twigs (AoGu_F1-3); A. taliensis female roots (Ata_F_R1-3), female stems (Ata_F_S1-3), female flowering twigs (Ata_F_1-3). **(D)** Conserved motifs architecture (MEME) showing retention and order of catalytic motifs; AofC24SMT1–1 and AtaC24SMT1–1 retain the motif set, supporting catalytic conservation across orthologs; **(E)** Exon-intron organization (longest isoforms per gene). Together, phylogenetic placement, motif integrity, and tissue expression nominate AofC24SMT1–1 and AtaC24SMT1–1 as lead candidates for experimental validation.

Comparative pathway reconstruction delineated the sterol biosynthesis routes in *Asparagus*, positioning C22SDs and C24SMTs at critical points between phytosterols and ERG synthesis (**Figure 1**; **Supplementary Figure 1**). These totals reflect post-filtering of HMM/BLAST hits by E-value (≤ 1e-5), sequence identity (≥ 40%) and alignment coverage (≥ 50%), retention of entries with the diagnostic domains (PF00067 for C22SDs; PF08498 for C24SMTs) verified in InterPro, consolidation to the longest isoform per locus, and phylogenetic placement with plant orthologs. Genome-wide screening identified 6 C22SD candidates in each species, as well as 4 and 7 C24SMT candidates in *A. officinalis* and *A. taliensis* (**Supplementary Table S1**). All candidates contained the expected diagnostic Pfam domains, validating assignment to sterol-modifying families. Neighbor-joining phylogenetic analysis (MEGA 11, 1000 bootstraps) resolved C22SDs into two clades: clade I containing AofC22SD1, AtaC22SD1 and AtaC22SD2 and clade II, comprising the remaining *Asparagus* C22SDs (**Figure 2A**). C24SMTs also grouped into two subfamilies, SMT1 and SMT2, consistent with canonical plant sterol diversification, suggesting functional divergence within *Asparagus* C24SMTs (**Figure 3A**).

Chromosomal distribution analysis showed dispersed localization across multiple chromosomes, with AtaC22SD4 the only candidate positioned on unanchored contigs (**Supplementary Figures 2C, D**). Together, these analyses provide a comprehensive catalog of sterol-modifying enzymes in *Asparagus* (*A. officinalis* and *A. taliensis*) and outlined their phylogenetic relationships with plant and fungal orthologs.

### 3.2 Conserved motif, gene structure, and cis-regulatory element analysis

Conserved motif analysis using MEME identified 10 recurrent motifs across the C22SD and C24SMT protein families (**Figures 2D**, **3D**; **Supplementary Tables S5A, B**). Within C22SDs, *AofC22SD1, AtaC22SD1*, and *AtaC22SD2* retained all 10 motifs in conserved order, indicating strong structural conservation. Other C22SDs lacked one or more motifs and/or displayed motif reordering, reflecting structural divergence among paralogs. C24SMTs exhibited greater heterogeneity in motif composition. SMT1 isoforms generally retained 7–9 motifs, whereas SMT2 isoforms carried 6–9 motifs. 4 predicted SMT2 proteins showed atypical motif arrangements, suggesting lineage-specific diversification within *Asparagus*.

Gene structure analysis using TBtools revealed variation in exon-intron architecture (**Figures 2E**, **3E**; **Supplementary Table S2**). In *A. officinalis*, 3 C22SD candidates (*AofC22SD1, AofC22SD2, and AofC22SD5)* had no introns, whereas the remaining genes contained multiple exons. *A. taliensis* presented a mix of intronless and intron-containing forms. For C24SMTs, *A. officinalis* genes were uniformly multi-exonic, while *A. taliensis* included both intronless (AtaC24SMT2-1) and multi-exonic members.

Cis-regulatory elements analysis of 2 kb promoter regions using PlantCARE identified numerous hormone-responsive motifs (abscisic acid, gibberellin, auxin, ethylene, MeJA, and salicyclic acid responsiveness), stress-responsive elements, and light responsive elements (**Supplementary Figures 2A, B**; **Supplementary Table S6**). The frequent occurrence of abscisic acid and MeJA-associated elements suggests potential regulatory involvement of sterol-modifying genes in response to abiotic and biotic stresses.

### 3.3 Expression profiling and co-expression network analysis

RNA-seq TPM matrices revealed tissue-biased expression of *C22SD* and *C24SMT* candidates across Rs, Ss, and Fs in both species (**Supplementary Table S7**). Several candidates showed broad expression across tissues (e.g., AofC22SD1, AofC24SMT1–1 and AtaC24SMT1-1), whereas others were tissue-biased (e.g., root-elevated AofC22SD3, AofC22SD6 and AofC24SMT2-2, flower-enriched AtaC24SMT2-3). These patterns suggest that some sterol-modifying enzymes perform general metabolic functions, while others fulfill tissue-specific roles potentially linked to localized stress or developmental cues.

**Figure 4:**
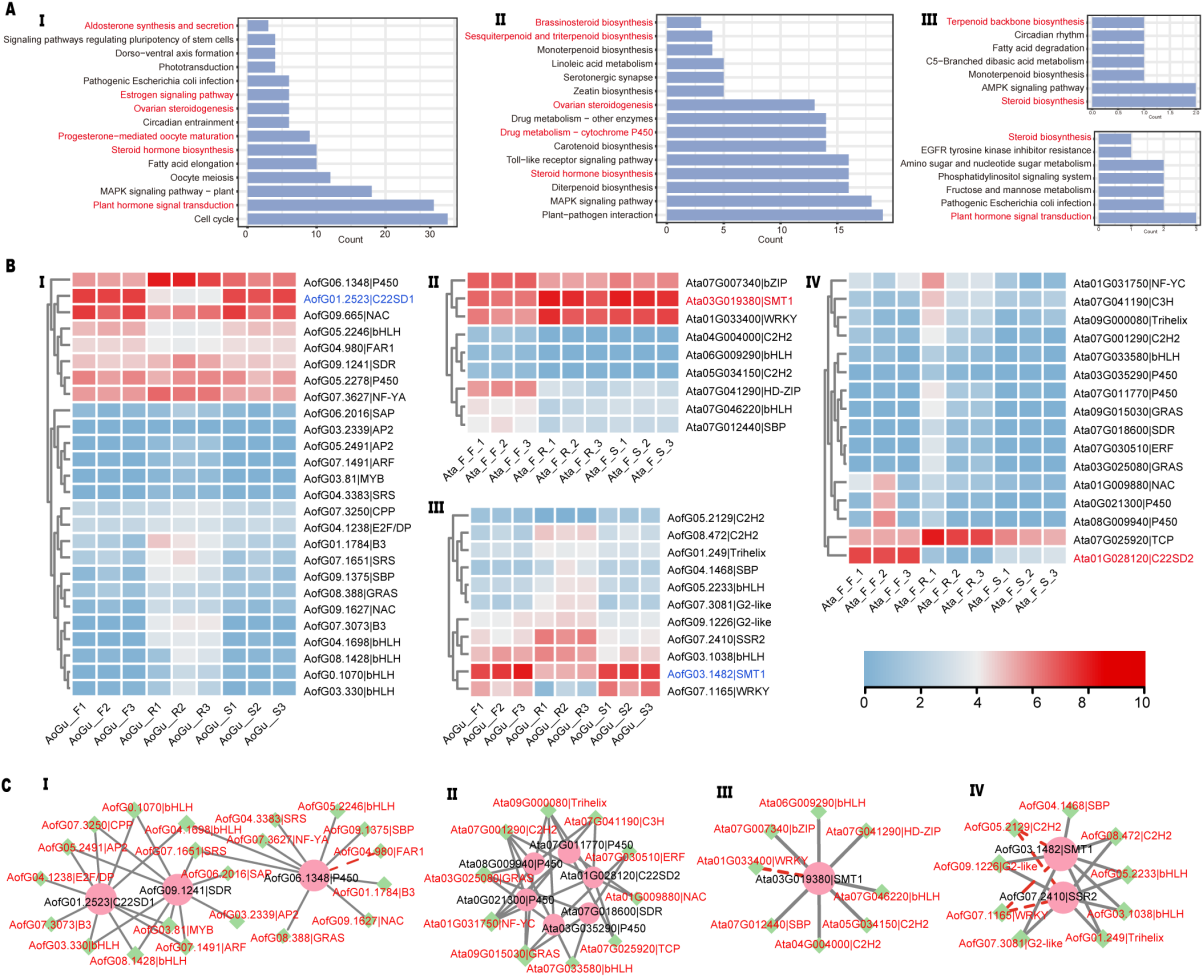
Exploratory co-expression context for C22SD and C24SMT candidates in *A officinalis* and *A taliensis.***(A)** Module KEGG summaries for the modules annotated to AofC22SD1 (I), AtaC22SD2 (II), AofC24SMT1-1 (III), and AtaC24SMT1-1 (IV). Steroid-related terms are highlighted; **(B)** Expression heatmaps of selected sterol-pathway genes from these modules across triplicate tissues: A. officinalis roots (AoGu_R1–3), stems (AoGu_S1–3), flowering twigs (AoGu_F1–3); A. taliensis female roots (Ata_F_R1–3), female stems (Ata_F_S1–3), female flowering twigs (Ata_F_1–3). A single-color scale applies to all panels; **(C)** Gene–TF co-variation maps (exploratory) for AofC22SD1 (I), AofC24SMT1-1 (II), AtaC24SMT1-1 (III), and AtaC22SD2 (IV). Sterol-modifying genes, TFs, and negative edges are shown as indicated in the diagram key.

Weighted gene co-expression network analysis (WGCNA) resolved 19 gene modules (GMs) in *A. officinalis* and 20 in *A. taliensis*. In *A. officinalis*, the brown module (BGM) contained *AofC22SD1*, and the orange module (OGM) contained *AofC24SMT1-1*. In *A. taliensis*, *AtaC22SD2* mapped to the red module (RGM) and *AtaC24SMT1-1*, to the cyan module (CGM). In *A. officinalis*, both the BGM and OGM were enriched in KEGG pathways related to steroid biosynthesis (**Figures 4A** i, iii, highlighted in red). In *A. taliensis*, the RGM and CGM modules were enriched in steroid metabolism and steroid hormone signaling (**Figures 4A** ii, iv). Heatmaps visualizing pathways-associated genes are shown for *A. taliensis* (**Figures 4B** i, iii; **Supplementary Table S7**; **S8**) and *A. officinalis* (**Figures 4B** ii, iv; **Supplementary Table S7, 8**).

Gene-TF associations within focal modules suggested coordinated regulation of sterol side-chain remodeling genes. In *A. officinalis*, multiple TFs were correlated with AofC22SD1 and a sterol dehydrogenase/reductase (SDR) homolog in the BGM; AofC24SMT1–1 and an *SSR2* homolog showed similar TF co-regulation in the OGM (**Figures 4C** i,iv). In *A. taliensis*, TFs co-varied with AtaC22SD2 and an SDR homolog in the RGM, while AtaC24SMT1–1 exhibited TF associations in the CGM (**Figures 4C** ii,iii). Promoter motif enrichment for module genes included TF-binding motifs also present in C22SD/C24SMT promoters (**Supplementary Table S8**), supporting the inferred regulatory links.

### 3.4 Protein structure predictions and molecular docking

3D modeling with Alphafold3 yielded high-confidence structural predictions for representative C22SD and C24SMT candidates. In both families, the core catalytic folds were conserved and closely resembled fungal orthologs, supporting evolutionary conservation of sterol side chain remodeling enzymes. For C22SDs, per-model ranking scores ranged from 0.57-0.95 in *A. officinalis* (lowest: AofC22SD6, 0.57; highest: AofC22SD1, 0.95) and from 0.90-0.95 in A. taliensis (highest: AtaC22SD2, 0.95) (**Supplementary Table S9A**). Alignment to *N. crassa* erg5 (NcC22SD) revealed the smallest RMSD revealed the smallest RMSD values for AofC22SD1 (1.50) and AtaC22SD2 (1.45), highlighting strong structural conservation. For C24SMTs, ranking scores ranged from 0.67-0.91 in A. officinalis (highest: AofC24SMT1-1, 0.91) and from 0.68-0.91 in A. taliensis (highest: AtaC24SMT1-1, 0.91). The lowest RMSD to NcSMT1 were observed for AofC24SMT1-1 (0.512) and AtaC24SMT1-1 (0.532) (**Supplementary Table S9B**).

**Figure 5:**
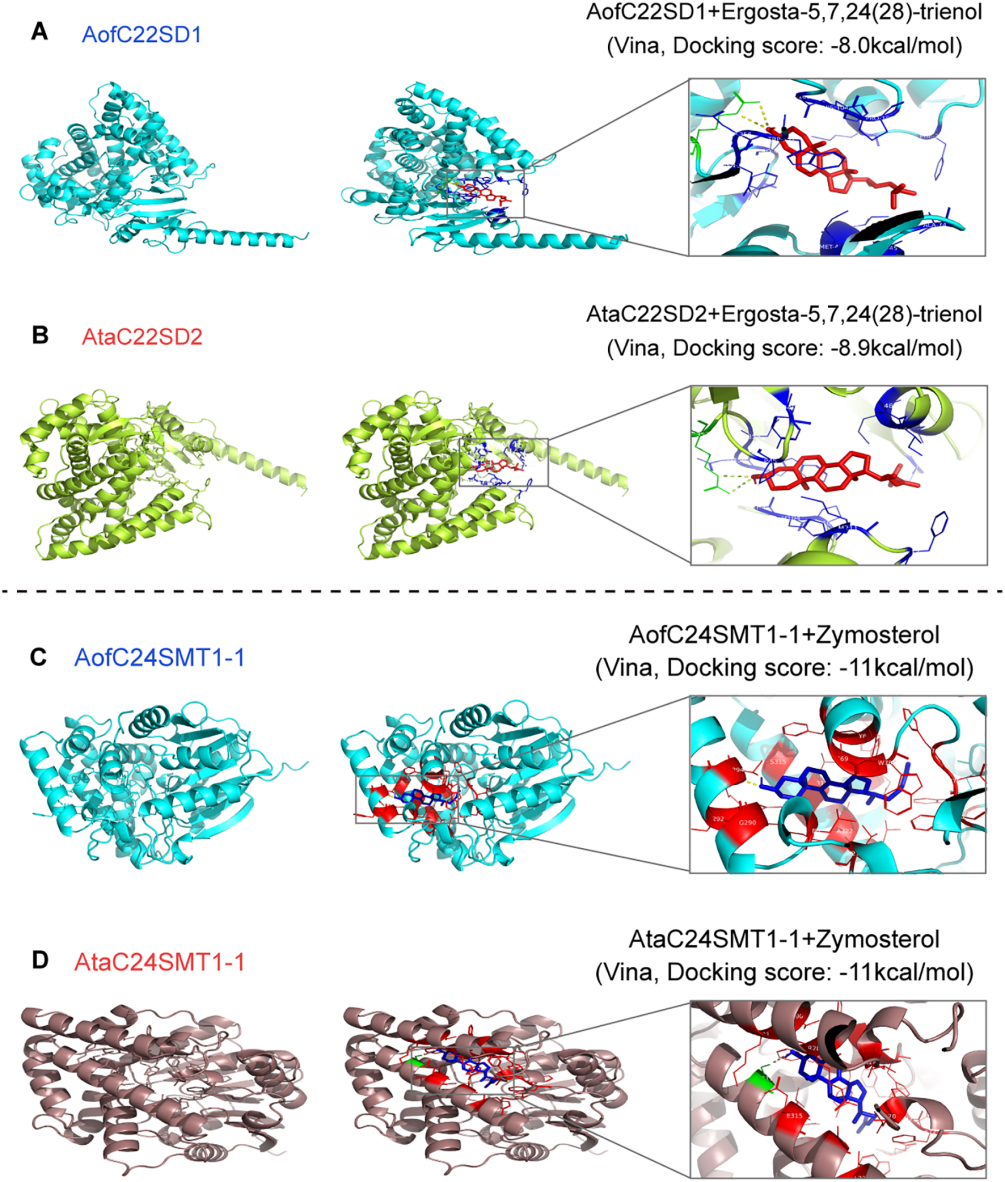
Structural modeling and docking of C22SD and C24SMT candidates with fungal sterol substrates. AlphaFold3-predicted 3D structures of representative C22SDs and C24SMTs with docked substrates illustrate conserved active-site geometry: **(A)** AofC22SD1 with ergosta-5,7,24(28)-trienol, **(B)** AtaC22SD2 with ergosta-5,7,24(28)-trienol, **(C)** AofC24SMT1–1 with zymosterol, and **(D)** AtaC24SMT1–1 with zymosterol. Docking analyses were performed qualitatively for pose ranking, and, together with conserved catalytic folds, support the functional assignment of these proteins as lead candidates for experimental validation.

Docking to predicted active-site cavities yielded favorable poses for the best-supported pairs. With ergosta-5,7,24(28)-trienol as the C22SD ligand, top Vina scores were -8.0 kcal/mol for AofC22SD1 and -8.9 kcal/mol for AtaC22SD2 (**Figures 5A, B**). For C24SMTs, docking with zymosterol produced scores of −11.0 kcal/mol for both AofC24SMT1–1 and AtaC24SMT1-1 (**Figures 5C, D**). Complete model metrics and docking results for all candidates are provided in **Supplementary Table S10** and **Supplementary Figure 3**-**6**. The combination of (i) high model confidence and low RMSD to fungal erg5/erg6 and (ii) consistently favorable docking for AofC22SD1/AtaC22SD2 and AofC24SMT1-1/AtaC24SMT1–1 supports functional conservation of the catalytic cores and nominates these four proteins as lead candidates for side-chain remodeling in *Asparagus*. Docking scores were used qualitatively for pose ranking, and the results provided structural rationale complementing heterologous complementation evidence for C22SD and C24SMT activity.

### 3.5 Δerg5/Δerg6 *N. crassa* disruptions and heterologous expression

**Figure 6:**
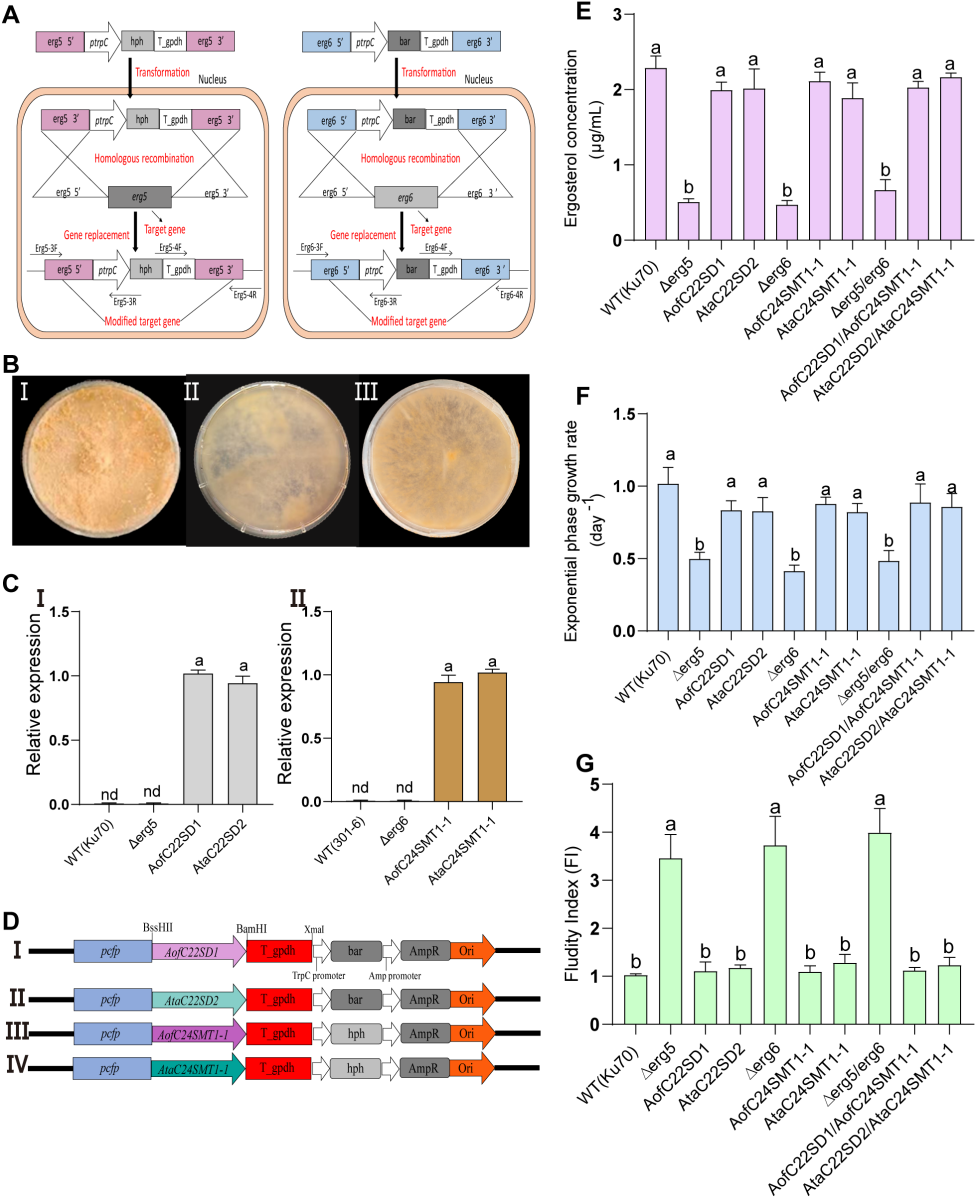
Functional complementation of Neurospora crassa erg5/erg6 mutants with Asparagus C22SD and C24SMT genes. **(A)** Targeted gene disruption strategy: (I) schematic replacement of erg5 with hph and (II) erg6 with bar markers by homologous recombination; **(B)** hyphal morphology: (I) wild type (Ku70) showing dense pigmentation, (II) mutant strains with thinner, paler hyphae, and (III) complemented strains partially restoring pigmentation and density; **(C)** RT-PCR verification of Asparagus C22SD and C24SMT expression in complemented strains; **(D)** schematic diagrams of chimeric gene constructs encoding (DI–DIV) AofC22SD1, AtaC22SD2, AofC24SMT1-1, and AtaC24SMT1–1 respectively, each driven by the cfp promoter and terminated with a gpdh terminator; **(E)** ergosterol concentrations in wild type, mutants, and complemented strains. Disruptions show ~70–80% reduction compared to WT, while complementation restores ergosterol to near WT levels; **(F)** exponential growth rates of wild type, mutants, and complemented strains, showing partial to near complete rescue upon complementation; **(G)** Plasma membrane fluidity index. Sterol depletion increases fluidity (WT normalized to 1.0), while complementation restores values toward WT. Collectively, these assays demonstrate that representative Asparagus C22SD and C24SMT genes functionally substitute for fungal erg5 and erg6, restoring sterol biosynthesis, growth, and membrane stability. Data are presented as mean ± SEM (n = 3). Different letters above bars indicate statistically significant differences at p < 0.05 (one-way ANOVA with Tukey’s *post hoc* test).

The fungal orthologs *erg5* and *erg6* were selected as hosts for heterologous testing based on their conserved roles in sterol side-chain remodeling. Disruption of these genes in N. crassa created defined backgrounds for evaluating the activity of Asparagus C22SD and C24SMT candidates. Gene replacement cassettes carrying selectable markers were introduced by homologous recombination, replacing the erg5 open reading frame with *hph* and erg6 with *bar* (**Figures 6A** i,ii). Primary transformants were recovered on the appropriate selective media. PCR verification confirmed correct locus replacement through marker amplification and locus-junction assays, establishing Δerg5 and Δerg6 single disruptions (**Supplementary Figures 7A**-I–IV).

To test combinatorial effects, single disruptions of opposite mating types were crossed to generate a Δerg5/erg6 double mutant. Segregants were recovered and screened by dual PCR to confirm the absence of both loci. These mutants provided genetic backgrounds for complementation experiments with Asparagus candidates.

Top-ranked C22SD (AofC22SD1, AtaC22SD2) and C24SMT (AofC24SMT1-1, AtaC24SMT1-1) candidates were cloned into fungal expression vectors and introduced into the corresponding disruption strains. A schematic of construct assembly and junction sites is provided in **Supplementary Figure S7**. Integration of transgenes was confirmed by PCR, and expression was verified by qRT-PCR using gene-specific primers (**Supplementary Figures 8A**VI–IX). Endogenous assays confirmed the absence of erg5 or erg6 transcripts in the disrupted strains (**Supplementary Figure 9**), while transgene assays detected expression of Asparagus C22SD and C24SMT genes in complemented lines (**Figure 6C**). These verifications establish the genetic and transcriptional states of the host backgrounds used for downstream functional analyses. Complementation in N. crassa indicates that the Asparagus enzymes catalyze the canonical reactions in a eukaryotic sterol pathway. These data do not establish physiological function in Asparagus tissues but provide a tractable activity assay pending in-planta tests.

Mating type verification by multiplex PCR distinguished parental strains (Mat A vs. Mat a) and confirmed successful generation of double-disrupted progeny (**Supplementary Figure 8A-X**). Collectively, these steps established a robust panel of single and double disruptions, along with complemented lines, for downstream analyses of sterol biosynthesis, growth, and membrane properties (**Supplementary Figure 8A-8V**; **Figure 8B**).

### 3.6 ERG quantification in wild-type, mutant, and complemented strains

ERG levels were quantified across N. crassa wild-type (Ku70), single knockouts (Δerg5, Δerg6), the double knockout (Δerg5/erg6), and complemented strains expressing selected Asparagus C22SD and C24SMT genes. GC–MS analysis revealed that disruption of erg5 or erg6 resulted in substantial reductions of ERG content, with concentrations of 0.507 μg/mL (Δerg5), 0.468 μg/mL (Δerg6), and 0.660 μg/mL (Δerg5/erg6), compared to 2.287 μg/mL in the wild type. These corresponded to ~71–79% reductions in sterol accumulation, with no significant differences among disrupted strains (**Figure 6E**). ERG in Δerg5/Δerg6 appeared numerically higher than in Δerg5 and Δerg6, but the differences were not significant. The small elevation likely reflects variation near the assay’s lower detection range and biomass normalization effects; all three mutants remained well below WT, and complementation restored ERG toward WT levels.

Complementation with *Asparagus* candidates restored ERG synthesis to near wild-type levels. Strains expressing AofC22SD1, AtaC22SD2, AofC24SMT1-1, or AtaC24SMT1-1, produced ERG concentrations ranging from 1.887 - 2.259 μg/mL, representing 82.5 - 98.9% of wild-type titers (**Figure 6E**). Double complementation lines (e.g., AofC22SD1/AofC24SMT1–1 and AtaC22SD2/AtaC24SMT1-1) exhibited comparable rescue efficiency, confirming that both C22SD and C24SMT enzymes can independently and jointly substitute for their fungal counterparts. Vector-only transformants showed no detectable increase in sterol levels or restoration of growth relative to the disrupted strains (data not shown).

GC–MS chromatograms validated the presence of sterol peaks corresponding to ergosterol standards, further confirming the biochemical recovery of ERG production in complemented strains (**Supplementary Figure 10**). These data provide direct evidence that Asparagus C22SD and C24SMT candidates functionally restore sterol biosynthesis in N. crassa disruption backgrounds, consistent with their predicted catalytic roles in sterol side-chain remodeling.

### 3.7 Phenotypic characterization of wild-type, mutant and complemented strains

#### 3.7.1 Hyphal morphology comparison

Qualitative assessment of hyphal morphology revealed striking differences among the *N. crassa* strains. The wild type exhibited densely packed, pigmented hyphae (**Figure 6Bi**), indicative of robust growth. In contrast, disruption strains (Δerg5, Δerg6, and Δerg5/erg6) showed visibly thinner and paler hyphae, reflecting impaired membrane integrity and growth potential (**Figure 6B** ii; **Supplementary Figure 8B-III and 8B-IV**). Complemented strains expressing selected *Asparagus* C22SD or C24SMT candidates partially restored hyphal density and pigmentation, aligning more closely with wild-type characteristics (**Figure 6B** ii; **Supplementary Figures 8B-V, 8B-VI**).

#### 3.7.2 Growth rate comparison

Quantitative measurements of exponential phase growth rates further supported the morphological observations. The wildtype reached an average growth rate of 1.001 g/L·day^-^¹, whereas Δerg5, Δerg6, and Δerg5/erg6 exhibited significantly reduced rates of 0.497, 0.413, and 0.483 g/L·day^-^¹, respectively. Complementation with AofC22SD1, AtaC22SD2, AofC24SMT1-1, or AtaC24SMT1–1 partially rescued growth, with rates ranging from 0.820–0.887 g/L·day^-^¹, representing 82 - 89% of wild-type levels (**Figure 6F**; **Supplementary Table 11**). These findings confirm that restored sterol biosynthesis translated into measurable improvements in fungal growth capacity.

#### 3.7.3 Membrane fluidity index comparison

Functional complementation also affected membrane properties. Disruption strains displayed markedly elevated membrane fluidity indices (3.458–3.988) compared to the wild-type baseline (1.00), consistent with sterol depletion altering membrane stability. Complemented strains showed values ranging from 1.095–1.280, reflecting near-complete restoration of wild-type membrane dynamics (**Figure 6G**).

Taken together, these phenotypic assays, spanning morphology, growth, and membrane function, demonstrate that *Asparagus* C22SD and C24SMT candidates functionally substitute for their fungal orthologs, rescuing defects in sterol biosynthesis and restoring key aspects of cellular physiology.

## 4 Discussion

Exploration of plant steroidal compounds, particularly SSs and BRs, has attracted increasing interest due to their roles in development and stress adaptation. SSs, derived from sapogenins with known bioactivities, are important for both agriculture and pharmaceuticals, whereas BRs regulate growth, reproduction, and stress tolerance (Chaudhuri et al., 2022; Nolan et al., 2019; Sonawane et al., 2016; Wang et al., 2022). The biosynthesis of these compounds is tightly regulated by sterol-modifying enzymes, including C-22SDs and C24SMTs, which occupy pivotal branch points in sterol metabolism (**Figure 1**; **Supplementary Figure S1**). In contrast to mammals and fungi, plants synthesize diverse phytosterols such as sitosterol, stigmasterol, campesterol, and CHOL, which contribute to membrane fluidity, stability and signaling (Valitova et al., 2016). These phytosterols also serve as precursors for BR and SS biosynthesis. C22SDs, as cytochrome P450 enzymes, catalyze double-bond insertion at the C22(23) position of sterol side chains (Aboobucker and Suza, 2019), while C24SMTs introduce side-chain methylations that diversify the sterol pool. Together with steroid side-chain reductases (SSRs) (Fellows et al., 2018), these enzymes coordinate biosynthetic branching to maintain balance between SS and BR production. Emerging evidence also positions membrane sterols as pleiotropic players in plant-microbe interactions, with sterol composition changes linked to signaling and defense (Der et al., 2024). To our knowledge, this study provides the first genome-wide catalog and, crucially, the first direct functional evidence for the catalytic activity of C22SD and C24SMT enzymes in any *Asparagus* species.

Previous studies in *Arabidopsis* (CYP710A1/2) and tomato (CYP710A11) established the role of C22SDs in directing stigmasterol biosynthesis and linking sterol remodeling to developmental processes (T. Morikawa et al., 2006; Whitaker and Gapper, 2008). Similarly, functional work on soybean SMT2 genes showed that sterol methylation influences membrane sterol composition (Neelakandan et al., 2010). Our study extends this body of knowledge by providing the first genome-wide identification and catalytic validation of C22SDs and C24SMTs in *Asparagus*. In doing so, we highlight the unique dual relevance of *Asparagus* as both a globally cultivated vegetable (*A. officinalis*) and a medicinal species with rich SS accumulation (*A. taliensis*), underscoring its importance as a model for exploring sterol remodeling in relation to both agriculture and phytochemistry (Cheng et al., 2023; Zeng et al., 2025). While our findings confirm catalytic activity of C22SDs and C24SMTs and suggest roles in sterol-mediated stress adaptation, we acknowledge that no direct abiotic stress assays were performed in planta. Stress relevance is therefore inferred, and future work would test these genes under stress conditions to validate their physiological contributions. Based on the conserved catalytic activity demonstrated here, we propose the following hypotheses for future validation in planta: (i) C24SMT up-modulation will increase 24-ethylsterols and proportionally constrain campesterol-derived BR flux, shifting growth–stress trade-offs under drought/salinity; (ii) C22SD up-modulation will elevate the stigmasterol/sitosterol ratio, altering plasma-membrane order and thereby buffering membrane fluidity under temperature/osmotic stress; and (iii) co-modulation of C22SD and C24SMT will rebalance sterol pools to influence downstream SS/BR branch flux. These hypotheses can be directly tested via transgenic or transient expression systems coupled with targeted sterolomics/saponinomics and standardized abiotic stress assays (Alessandro Cabianca et al., 2021).

Expression and promoter analyses indicate that sterol-modifying genes in Asparagus are positioned to respond to stress-related hormonal cues. Enrichment of ABA- and MeJA- responsive cis-elements, together with tissue bias (e.g., root-elevated candidates), points to context-dependent regulation under drought/salinity (ABA) and defense signaling (JA). Mechanistically, C24SMTs partition flux between 24-methyl (campesterol) and 24-ethyl (sitosterol) sterols, thereby influencing both membrane sterol composition and the availability of campesterol as a brassinosteroid (BR) precursor. C22SD controls the sitosterol to stigmasterol step, modulating the sitosterol/stigmasterol ratio that shapes plasma-membrane order, nanodomain organization, and receptor/transporter behavior. These relationships support the hypothesis that context-specific tuning of C24SMT activity could increase campesterol-derived BR signaling to support adaptive growth, whereas C22SD up-modulation could adjust sterol ratios to stabilize membranes and interface with stress/defense pathways.

Transcriptomic analyses revealed high expression of selected C22SDs and C24SMTs in photosynthetic and reproductive tissues (**Figures 2B, C**, **3B, C**; **Supplementary Table S7**). Co-expression modules linked these genes to other sterol pathway enzymes and TFs from B3, ARF, AP2, and bHLH families (**Figures 4A–C**). Promoter analysis identified cis-regulatory elements responsive to auxin, ABA, MeJA, and light (**Supplementary Figures S2A, B**; **Supplementary Table S6**), implying transcriptional fine-tuning of BR-SS balance under hormonal and environmental cues. The frequent occurrence of ABA-responsive elements is consistent with ABA’s central role in drought and salinity signaling, while MeJA-responsive elements align with jasmonate-mediated defense pathways. Together with the observed tissue biases, these features support a model in which sterol-modifying enzymes interface with hormone-dependent stress adaptation and defense signaling in *Asparagus*.

Functional assays employed *N. crassa* as a heterologous host due to transformation barriers in Asparagus. *N. crassa* was selected due to its efficient transformation system and conserved sterol biosynthetic pathway (Borkovich et al., 2004; Davis and Perkins, 2002; Ruger-Herreros and Corrochano, 2020). While our results demonstrate successful functional validation in *N. crassa*, future work should extend these findings to *Asparagus* or related plants to confirm their physiological relevance. Disruption of erg5 and erg6 or both yielded sterol-deficient mutants with ERG reductions of 77.8-79.5% compared to wild-type, along with impaired growth and abnormal morphology (**Figures 6E, F**). Interestingly, the Δerg5/erg6 double mutant displayed a slightly higher ergosterol concentration than either single mutant. However, this difference was not statistically significant (**Figure 6E**). Although not statistically significant, this small elevation could reflect feedback regulation of upstream sterol synthesis, flux redistribution among sterol intermediates that partially restores ERG-like species detectable by our derivatization/GC–MS method, or normalization effects linked to biomass differences; resolving these alternatives will require targeted isotopic flux tracing and enzyme-level assays.

Complementation with single or double expression constructs of AofC22SD1, AtaC22SD2, AofC24SMT1–1 and AtaC24SMT1–1 restored ERG production to near wild-type titers (1.858-2.259 µg/mL), accompanied by recovery of hyphal morphology and membrane fluidity (**Figures 6B, G**; **Supplementary Figure S10**). These results provide direct functional evidence that *Asparagus* C22SDs and C24SMTs substitute for fungal orthologs, reinforcing their catalytic activity in sterol biosynthesis. Robust rescue in *N. crassa* supports strong conservation and functional portability of *Asparagus* C24SMTs/C22SDs across sterol backbones.

Sterol remodeling directly influences membrane biophysics, ensuring stability under temperature extremes, salinity, and drought (Aboobucker and Suza, 2019). In fungal complementation assays, sterol depletion increased membrane fluidity, while expression of *Asparagus* enzymes restored stability (**Figure 6G**). This finding parallels reports that BRs enhance stress tolerance through hormonal signaling cascades (Wang et al., 2022), whereas SSs function as antimicrobial and deterrent metabolites in biotic defense (Shakeel et al., 2025). The functional validation of core *Asparagus* enzymes, combined with expression in stress-sensitive tissues, supports a model in which C22SDs and C24SMTs maintain sterol pools that underpin both growth-stress trade-offs and chemical defense.

Validated catalytic functions designate C22SDs and C24SMTs as promising targets for translational applications. Potential strategies include engineering sterol ratios to enhance membrane rigidity under abiotic stress, modulating BR biosynthetic flux for adaptive growth regulation, or augmenting SS production to reinforce antimicrobial defenses. Future work involving enzyme assays with plant substrates, targeted metabolomics, and TF-promoter interaction studies will further refine the link between sterol gene variation, metabolic branching, and stress resilience. Functional validation in heterologous systems establishes a methodological platform for dissecting sterol genes in species with limited genetic resources, positioning *Asparagus* as a model for SS research. These enzymes enable multiple application routes: (i) rebalance sterol ratios to enhance membrane stability under drought, salinity, and temperature stress; (ii) tuning BR flux to optimize growth–stress trade-offs; and (iii) elevate steroidal saponin (SS) accumulation for antimicrobial defense and phytochemical value.

## 5 Conclusion

C22SDs and C24SMTs were identified and validated as conserved sterol-modifying enzymes in *A. officinalis* and *A. taliensis*. Structural modeling, expression profiling, and promoter analyses revealed functional conservation and regulatory integration with stress-responsive networks. Heterologous complementation in *N. crassa* confirmed catalytic activity, restoring sterol production, growth, and membrane stability. Together, these findings demonstrate that C22SDs and C24SMTs act at pivotal branch points directing sterol flux toward BR and SS biosynthesis, linking sterol remodeling to abiotic stress tolerance and biotic defense. Looking forward, these functions provide clear opportunities for crop improvement. Key applications include rebalancing sterol ratios for membrane stability under stress, tuning BR flux to optimize growth/stress trade-offs, and enhancing SS production for antimicrobial defense and phytochemical value. Beyond *Asparagus*, the validation pipeline established here offers a general framework for accelerating stress-resilient and metabolically enhanced crop development in species with limited transformation systems.

## Data Availability

The RNA-seq datasets analyzed in this study are publicly available at the China National Center for Bioinformation (CNCB). Data for *Asparagus officinalis* is deposited under BioProject PRJCA011702, and data for *Asparagus taliensis* are deposited under BioProject PRJCA011431. Additional data supporting the findings of this study are available within the article and its **Supplementary Material**.

## Supplementary material

Supplementary Figure 1 Schematic representation of sterol biosynthesis in plants and *Neurospora crassa*. The pathway is organized into four modules. The upper uncolored section represents the shared early steps from acetyl-CoA to 2,3-oxidosqualene via the mevalonate (MVA) pathway. In plants (*A. officinalis* and *A. taliensis*), downstream branches lead to steroidal saponins (SSs, gray) and brassinosteroids (BRs, pink). In N. crassa, the pathway proceeds through lanosterol to ergosterol (ERG, blue). Genes selected for functional validation in this study, C22SD and SMT1 and their known fungal orthologs, erg5 and erg6, are shown in orange and red respectively.

Supplementary Figure 2 Predicted CRE and chromosomal distribution of C22SD and C24SMT genes in *Asparagus*. **(A)** Heatmaps of predicted CREs within 2 kb promoter regions of **(A)** C22SDs and **(B)** C24SMTs genes. Elements were grouped into 3 functional categories: phytohormone responsive, stress responsive and growth related. Abundance values were normalized and scaled from 0 (no abundance) to 1(maximum abundance) using TBtools. Key hormone responsive CREs including abscisic acid (ABA), auxin (IAA), ethylene (ERE), gibberellin (GA), methyl jasmonate (MeJA) and salicylic acid (SA); **(C, D)** chromosomal distribution of **(C)** C22SDs and **(D)** C24SMTs genes in A. officinalis (green) and A. taliensis (turquoise blue). Chromosome lengths are shown to scale. Representative genes are highlighted in blue (*A. officinalis*) and red (*A. taliensis*).

Supplementary Figure 3 **(A)** The 3D structures of the 5 remaining predicted *A. officinalis* C22SDs; **(B)** molecular docking analysis of the candidates with the ligand ergosta-5,7,24(28)-trienol, showing their respective docking scores.

Supplementary Figure 4 **(A)** The 3D structures of the 5 remaining predicted *A. taliensis* C22SDs; **(B)** molecular docking analysis of the candidates with the ligand ergosta-5,7,24(28)-trienol, showing their respective docking scores.

Supplementary Figure 5 **(A)** The 3D structures of the 3 remaining predicted *A. officinalis* C24SMTs; **(B)** molecular docking analysis of the candidates with the ligand zymosterol, showing their respective docking scores.

Supplementary Figure 6 **(A)** The 3D structures of the 6 remaining predicted *A. taliensis* C24SMTs; **(B)** molecular docking analysis of the candidates with the ligand zymosterol, showing their respective docking scores.

Supplementary Figure 7 Step-by-step construction of the AtaC22SD expression vector. Linearized maps show assembly from the pcfp backbone to pAtaC22SD via BssHII/BamHI/XmaI digestions and ligations. PCR-amplified AtaC22SD CDS and the T_gpdh terminator were inserted at the indicated junctions; enzyme sites and fragment sizes (bp) are annotated on each step. Figure generated in SnapGene (Dotmatics).

Supplementary Figure 8 Molecular confirmation and phenotypic characterization of Neurospora crassa transformants expressing Asparagus C22SD and C24SMT genes. **(A)** PCR validation of transformants: (I) Δerg5, (II) Δerg6, (III, IV) Δerg5Δerg6 double mutants, (V) mating type confirmation, (VI) AofC22SD1, (VII) AofC24SMT1-1, (VIII) AtaC22SD2, (IX) AtaC24SMT1-1, and (X) co-transformants (AofC22SD1/AofC24SMT1-1) in A. officinalis and (AtaC22SD2/AtaC24SMT1-1) A. taliensis. M: DNA marker; C: wild-type; “+” and “–”: positive and negative controls; numbers denote independent transformants. **(B)** Phenotypic assays. (I, II) Crossing plate comparison showing (I) unsuccessful and (II) successful ascospore development. Colony morphologies of (III, IV) disrupted and (V, VI) complemented strains grown under standard culture conditions.

Supplementary Figure 9 Transcript validation of *N. crassa* ergosterol biosynthesis gene disruptions. Relative expression levels of target genes in wild-type and deletion strains: **(A)***erg5* expression in WT (Ku70) and Δerg5; **(B)***erg6* expression in WT (301-6) and Δerg6. Expression was normalized to wild-type controls (set to 1.0). Bars represent as means (n = 3) of the strains. “nd” indicates no detectable expression.

Supplementary Figure 10 GC–MS chromatograms validating sterol pathway disruption and complementation in *N. crassa* WT (Ku70), Δerg5, Δerg6, Δerg5/Δerg6, and complemented strains expressing *Asparagus officinalis* or *A. taliensis* C22SD and C24SMT genes. Complemented strains include Δerg5+*AofC22SD1*, Δerg5+*AtaC22SD2*, Δerg6+*AofC24SMT1-1*, Δerg6+*AtaC24SMT1-1*, Δerg5/Δerg6+*AofC22SD1/AofC24SMT1-1*, and Δerg5/Δerg6+*AtaC22SD2/AtaC24SMT1-1*. Diagnostic peaks corresponding to sterol products are boxed in red. One spectrum is shown per condition; all replicates yielded highly similar fragmentation patterns
