# Supplementary figures and images for: Genome-wide identification and functional validation of sterol C-22 desaturases and C-24 methyltransferases in Asparagus officinalis and Asparagus taliensis
Source: Front Plant Sci. 2025 Nov 18;16:1690526. doi: 10.3389/fpls.2025.1690526 (PMC12671487; doi:10.3389/fpls.2025.1690526)

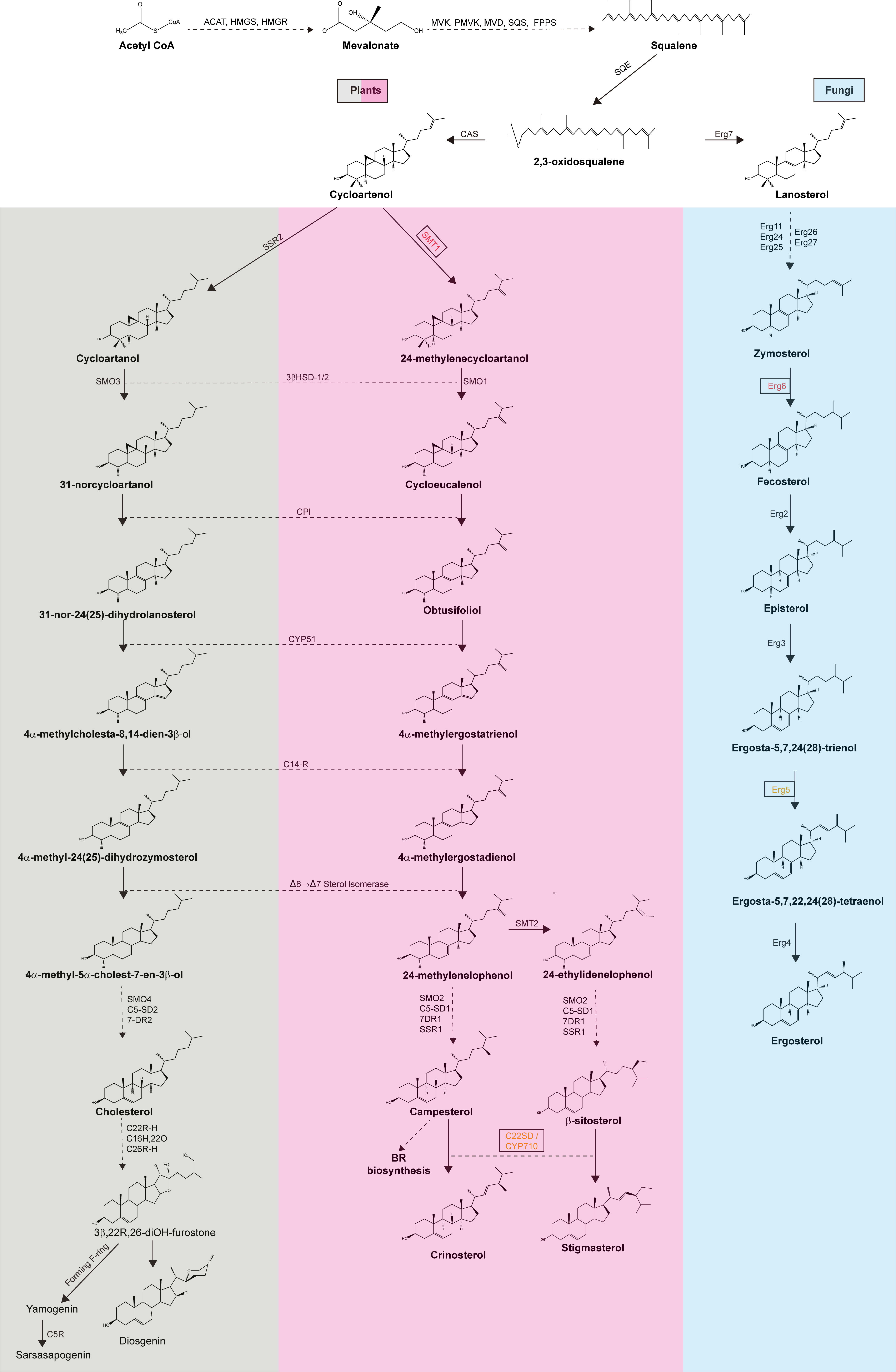

Supplement: Supplementary Figure 1 — Schematic representation of sterol biosynthesis in plants and Neurospora crassa. The pathway is organized into four modules. The upper uncolored section represents the shared early steps from acetyl-CoA to 2,3-oxidosqualene via the mevalonate (MVA) pathway. In plants (A. officinalis and A. taliensis), downstream branches lead to steroidal saponins (SSs, gray) and brassinosteroids (BRs, pink). In N. crassa, the pathway proceeds through lanosterol to ergosterol (ERG, blue). Genes selected for functional validation in this study, C22SD and SMT1 and their known fungal orthologs, erg5 and erg6, are shown in orange and red respectively. [file Image1.tif]

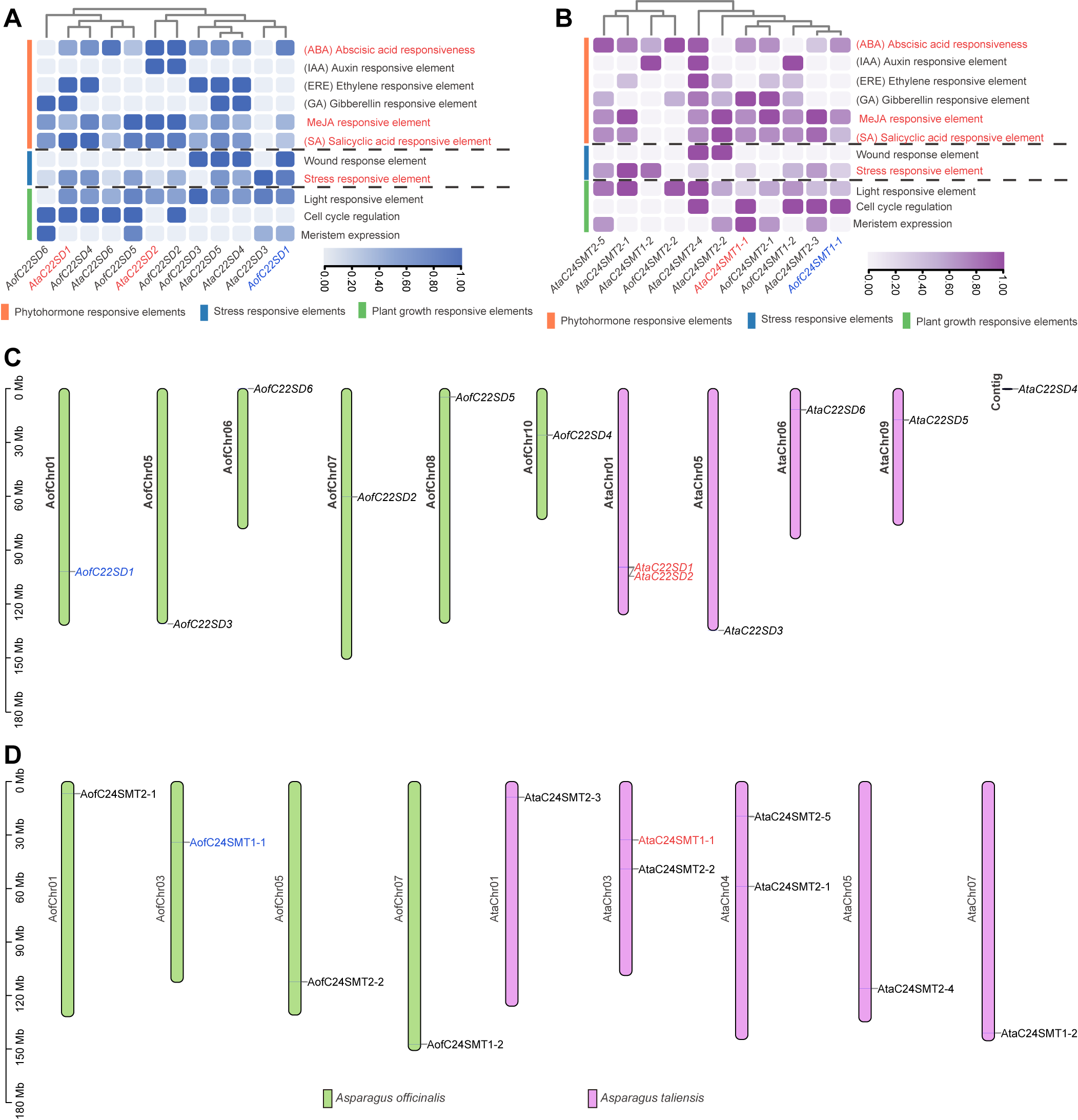

Supplement: Supplementary Figure 2 — Predicted CRE and chromosomal distribution of C22SD and C24SMT genes in Asparagus. (A) Heatmaps of predicted CREs within 2 kb promoter regions of (A) C22SDs and (B) C24SMTs genes. Elements were grouped into 3 functional categories: phytohormone responsive, stress responsive and growth related. Abundance values were normalized and scaled from 0 (no abundance) to 1(maximum abundance) using TBtools. Key hormone responsive CREs including abscisic acid (ABA), auxin (IAA), ethylene (ERE), gibberellin (GA), methyl jasmonate (MeJA) and salicylic acid (SA); (C, D) chromosomal distribution of (C) C22SDs and (D) C24SMTs genes in A. officinalis (green) and A. taliensis (turquoise blue). Chromosome lengths are shown to scale. Representative genes are highlighted in blue (A. officinalis) and red (A. taliensis). [file Image2.tif]

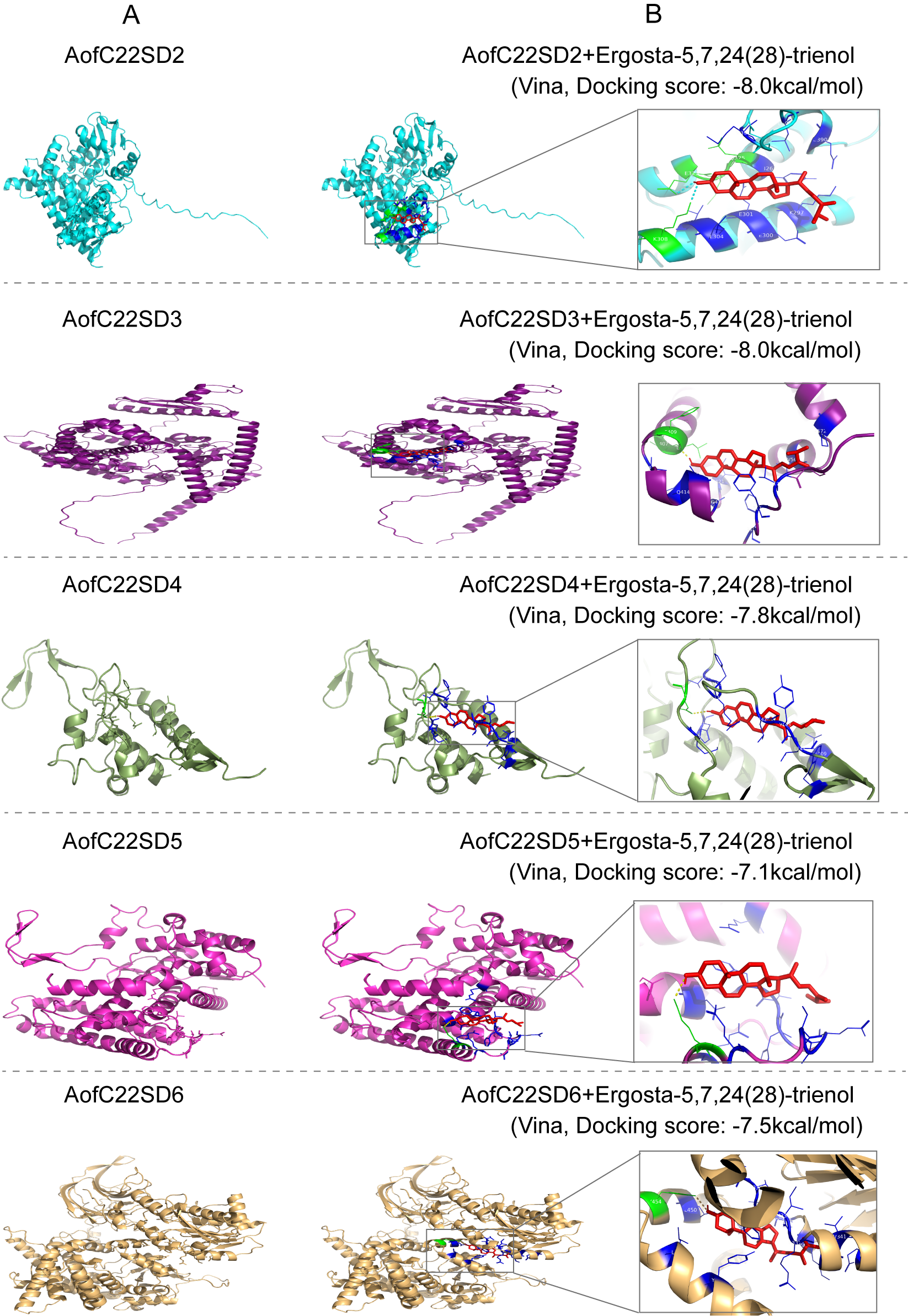

Supplement: Supplementary Figure 3 — (A) The 3D structures of the 5 remaining predicted A. officinalis C22SDs; (B) molecular docking analysis of the candidates with the ligand ergosta-5,7,24(28)-trienol, showing their respective docking scores. [file Image3.tif]

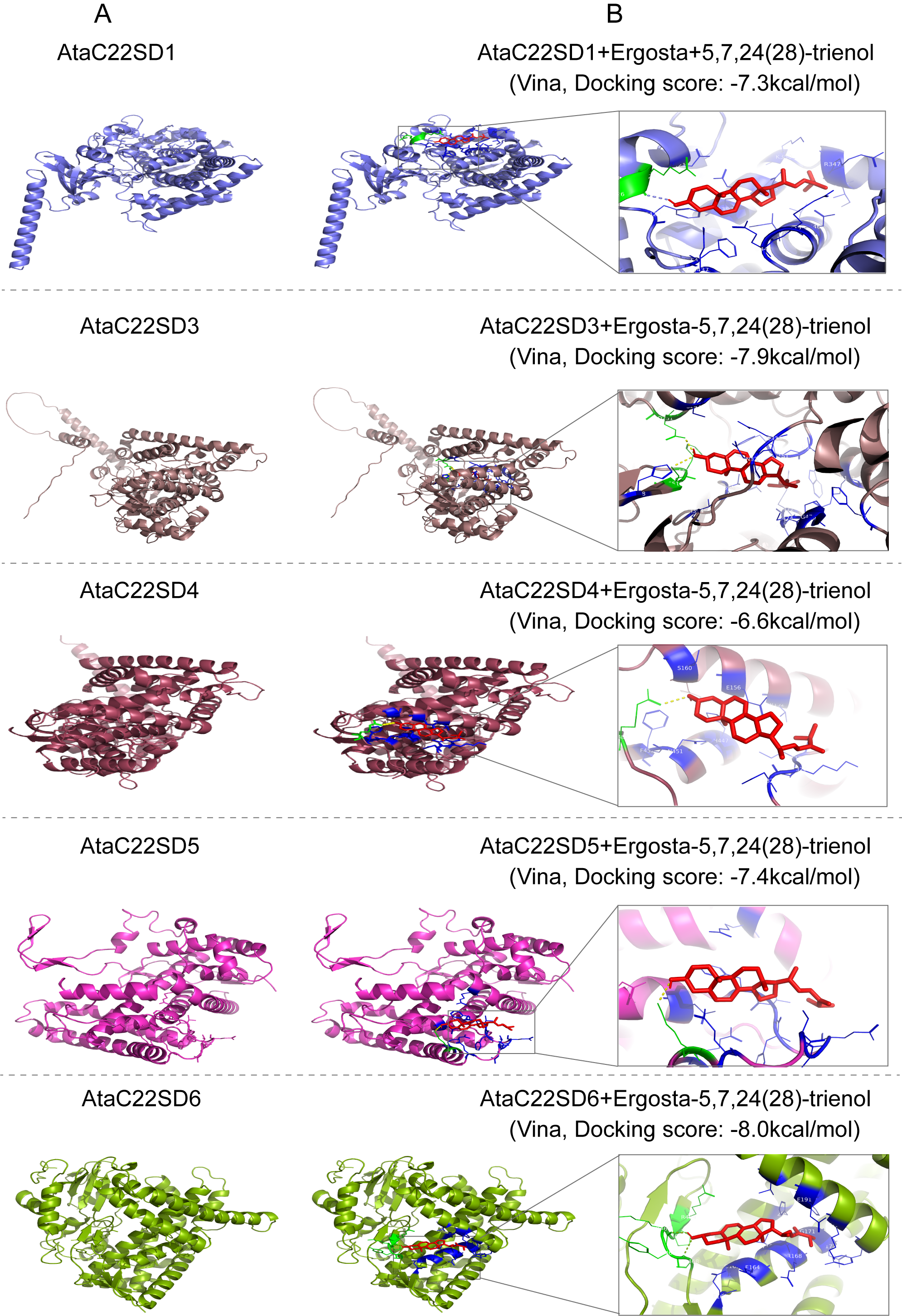

Supplement: Supplementary Figure 4 — (A) The 3D structures of the 5 remaining predicted A. taliensis C22SDs; (B) molecular docking analysis of the candidates with the ligand ergosta-5,7,24(28)-trienol, showing their respective docking scores. [file Image4.tif]

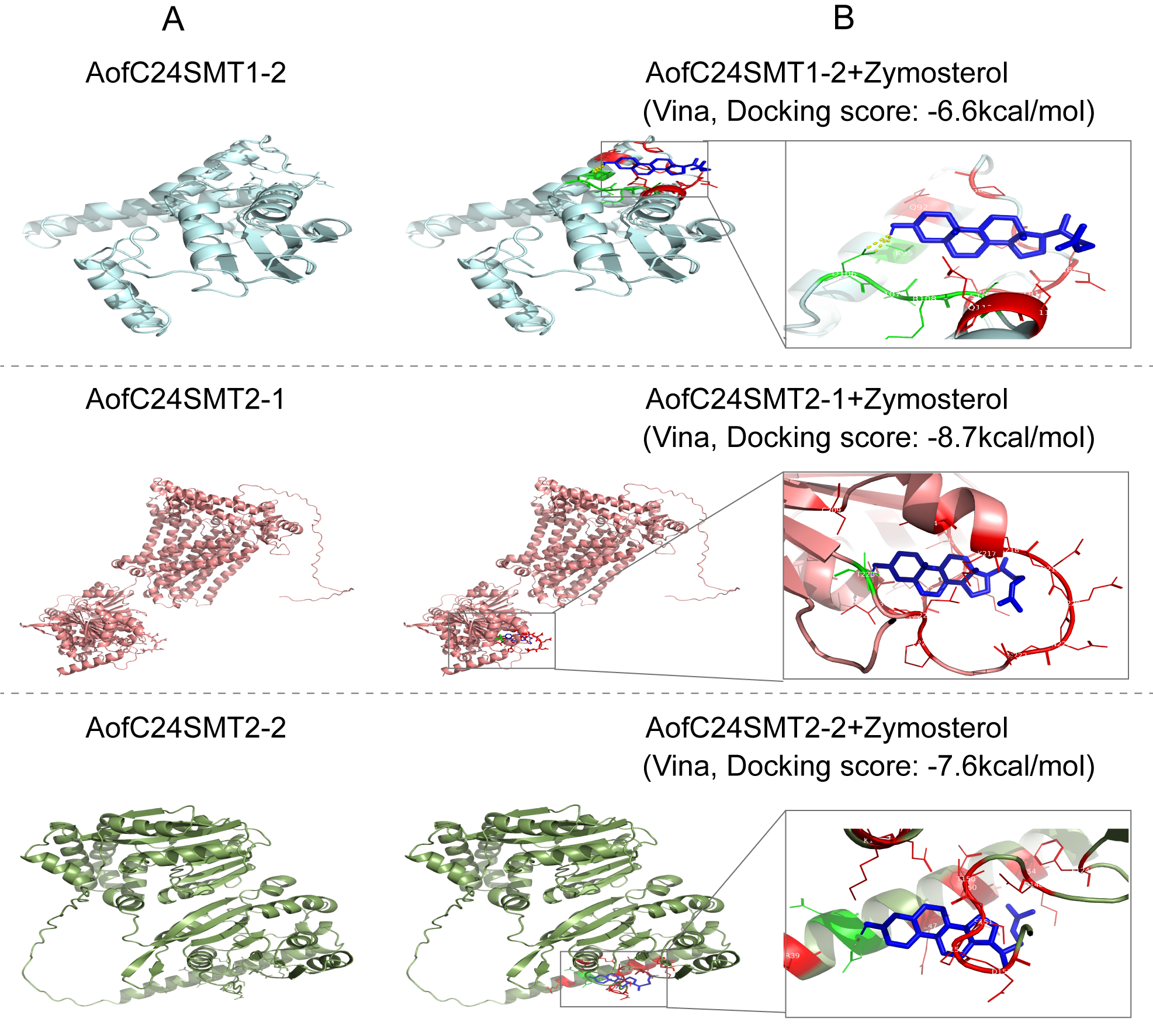

Supplement: Supplementary Figure 5 — (A) The 3D structures of the 3 remaining predicted A. officinalis C24SMTs; (B) molecular docking analysis of the candidates with the ligand zymosterol, showing their respective docking scores. [file Image5.tif]

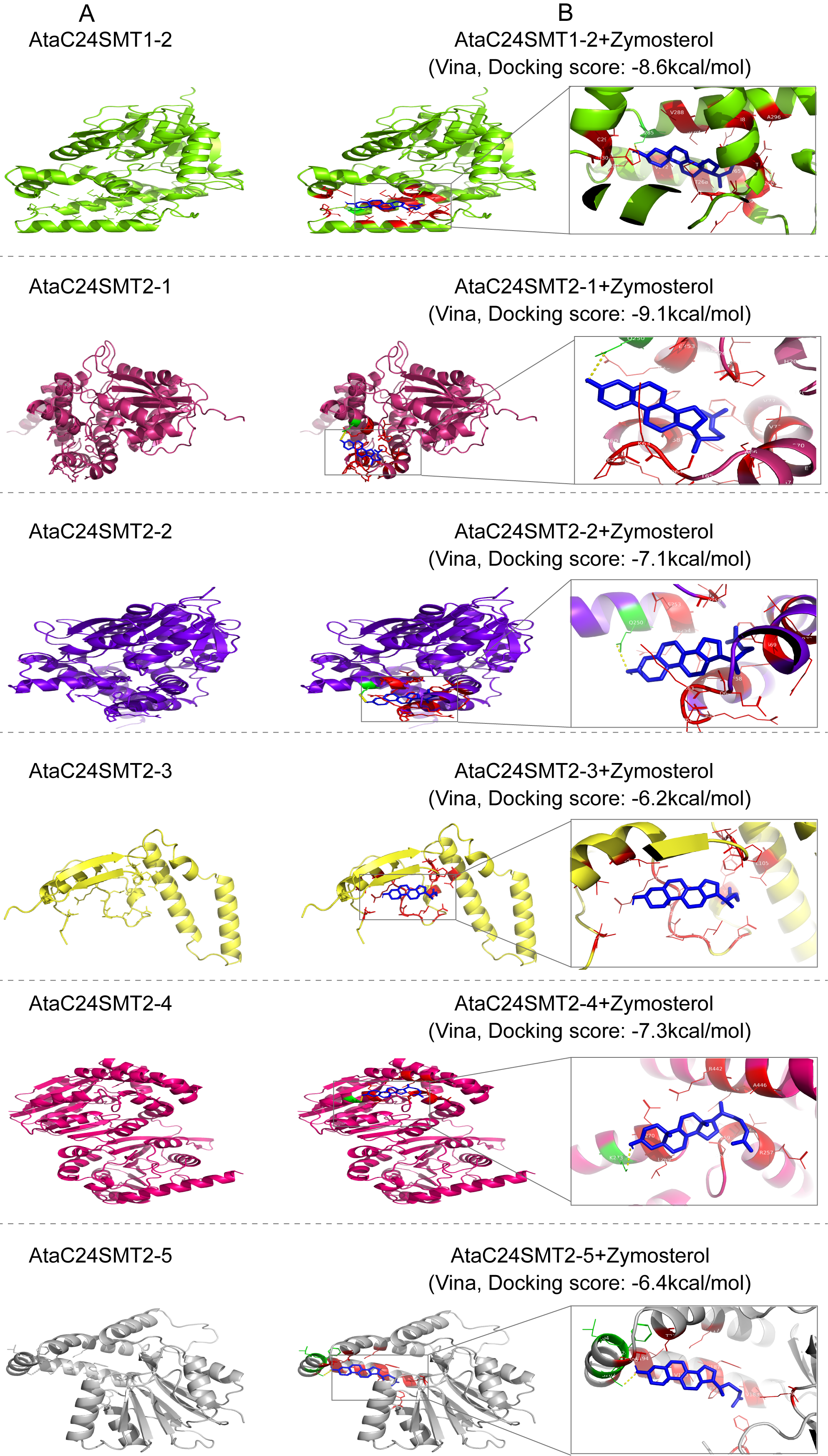

Supplement: Supplementary Figure 6 — (A) The 3D structures of the 6 remaining predicted A. taliensis C24SMTs; (B) molecular docking analysis of the candidates with the ligand zymosterol, showing their respective docking scores. [file Image6.tif]

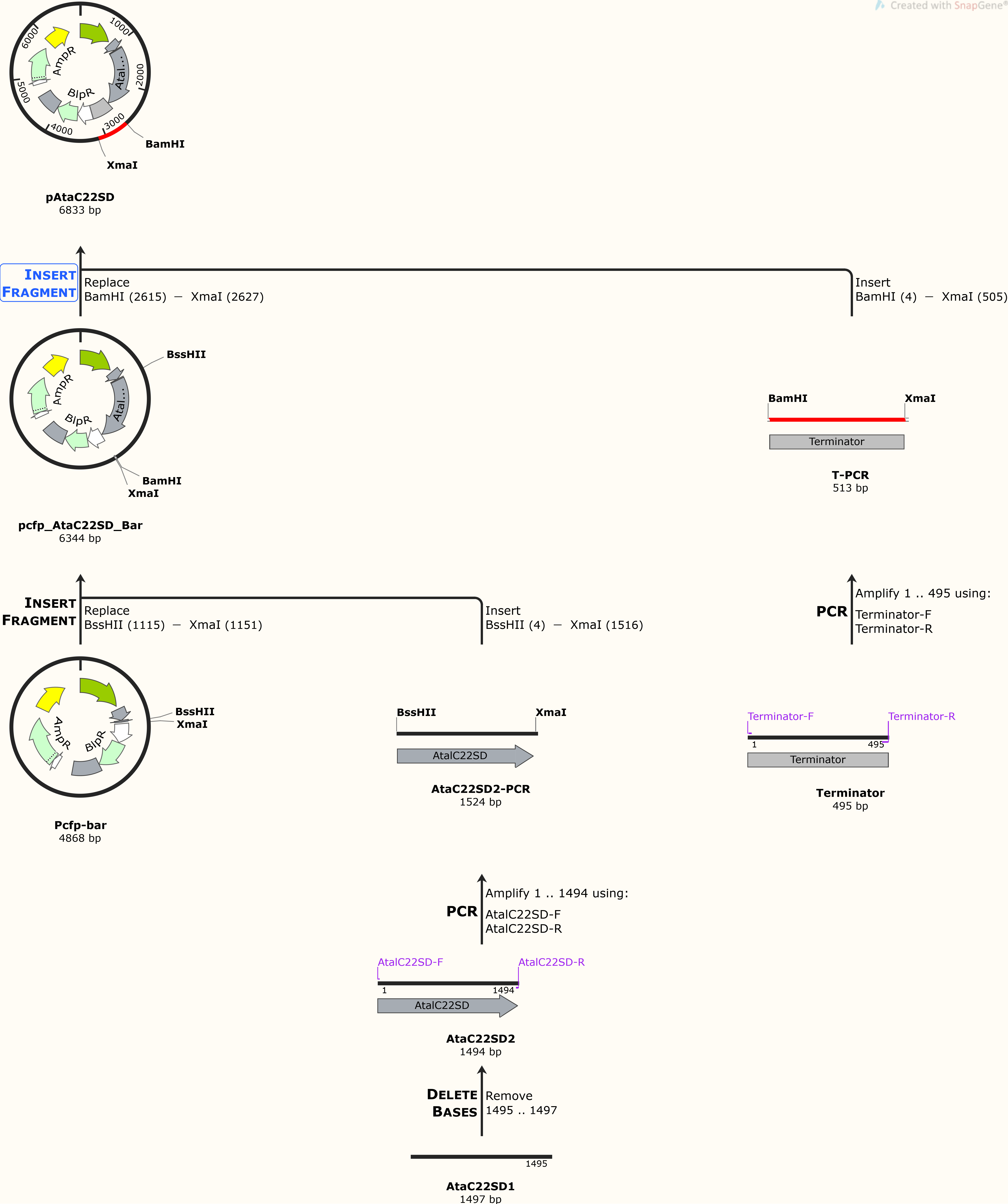

Supplement: Supplementary Figure 7 — Step-by-step construction of the AtaC22SD expression vector. Linearized maps show assembly from the pcfp backbone to pAtaC22SD via BssHII/BamHI/XmaI digestions and ligations. PCR-amplified AtaC22SD CDS and the T_gpdh terminator were inserted at the indicated junctions; enzyme sites and fragment sizes (bp) are annotated on each step. Figure generated in SnapGene (Dotmatics). [file Image7.tiff]

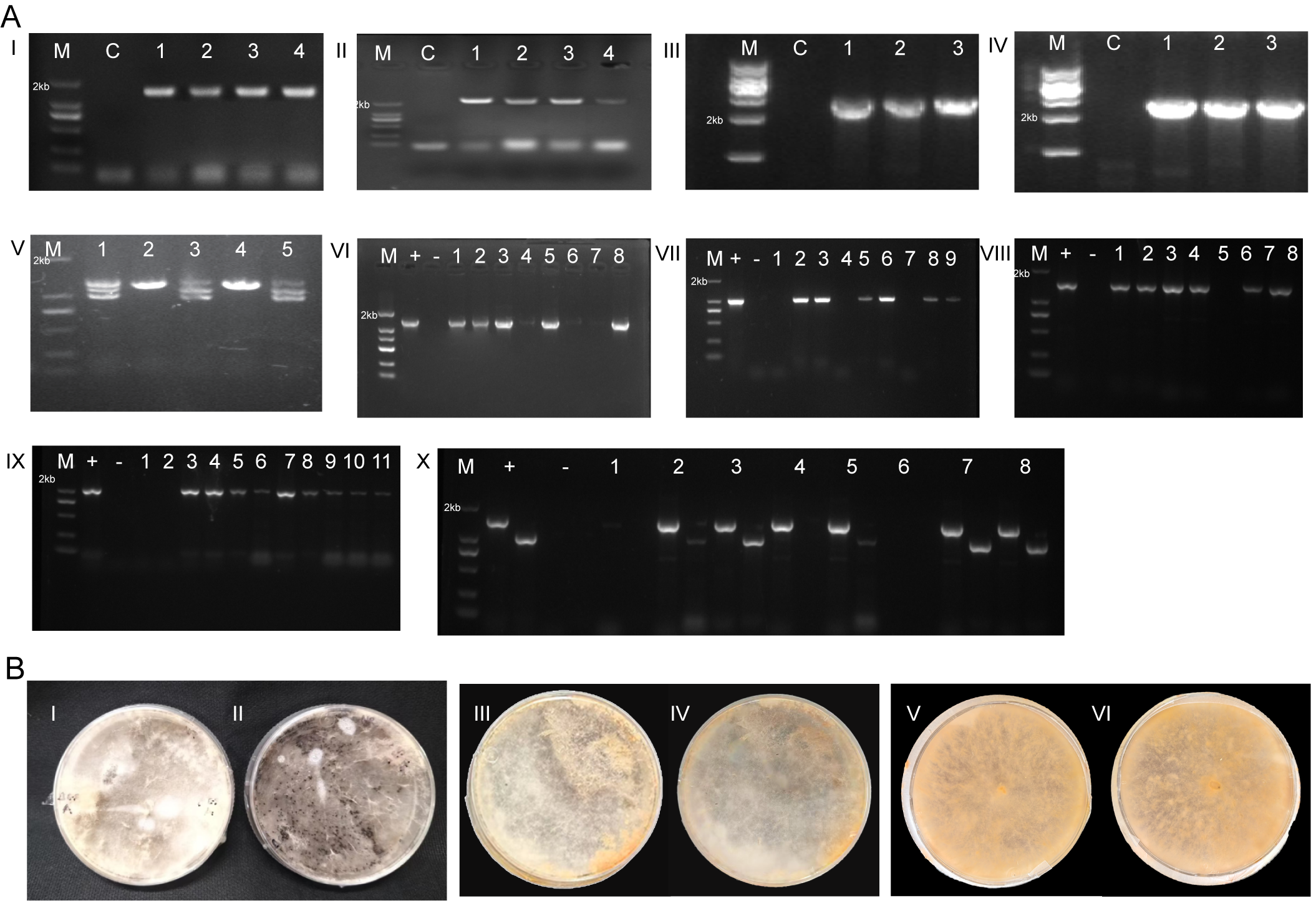

Supplement: Supplementary Figure 8 — Molecular confirmation and phenotypic characterization of Neurospora crassa transformants expressing Asparagus C22SD and C24SMT genes. (A) PCR validation of transformants: (I) Δerg5, (II) Δerg6, (III, IV) Δerg5Δerg6 double mutants, (V) mating type confirmation, (VI) AofC22SD1, (VII) AofC24SMT1-1, (VIII) AtaC22SD2, (IX) AtaC24SMT1-1, and (X) co-transformants (AofC22SD1/AofC24SMT1-1) in A. officinalis and (AtaC22SD2/AtaC24SMT1-1) A. taliensis. M: DNA marker; C: wild-type; “+” and “–”: positive and negative controls; numbers denote independent transformants. (B) Phenotypic assays. (I, II) Crossing plate comparison showing (I) unsuccessful and (II) successful ascospore development. Colony morphologies of (III, IV) disrupted and (V, VI) complemented strains grown under standard culture conditions. [file Image8.tif]

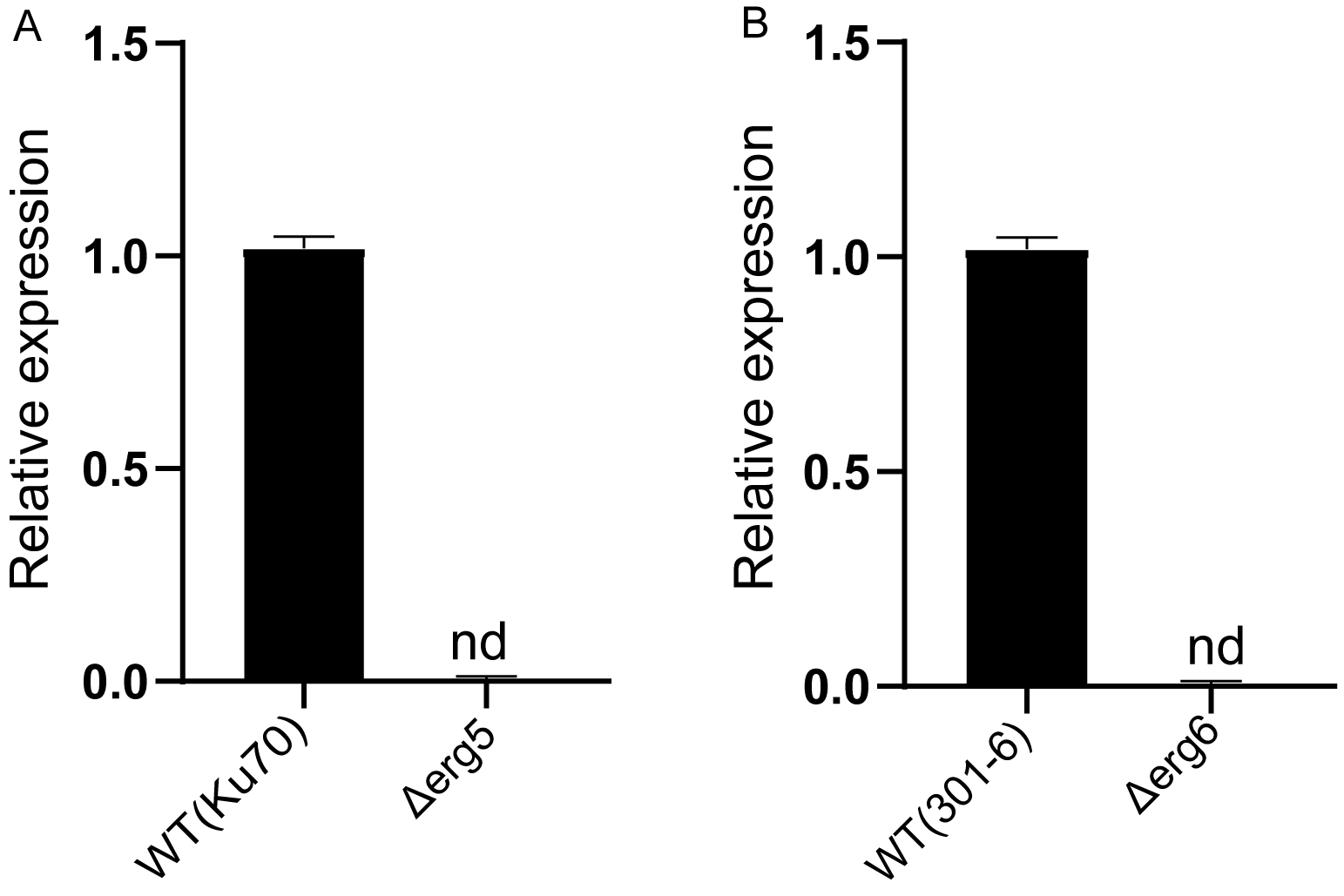

Supplement: Supplementary Figure 9 — Transcript validation of N. crassa ergosterol biosynthesis gene disruptions. Relative expression levels of target genes in wild-type and deletion strains: (A)erg5 expression in WT (Ku70) and Δerg5; (B)erg6 expression in WT (301-6) and Δerg6. Expression was normalized to wild-type controls (set to 1.0). Bars represent as means (n = 3) of the strains. “nd” indicates no detectable expression. [file Image9.tif]

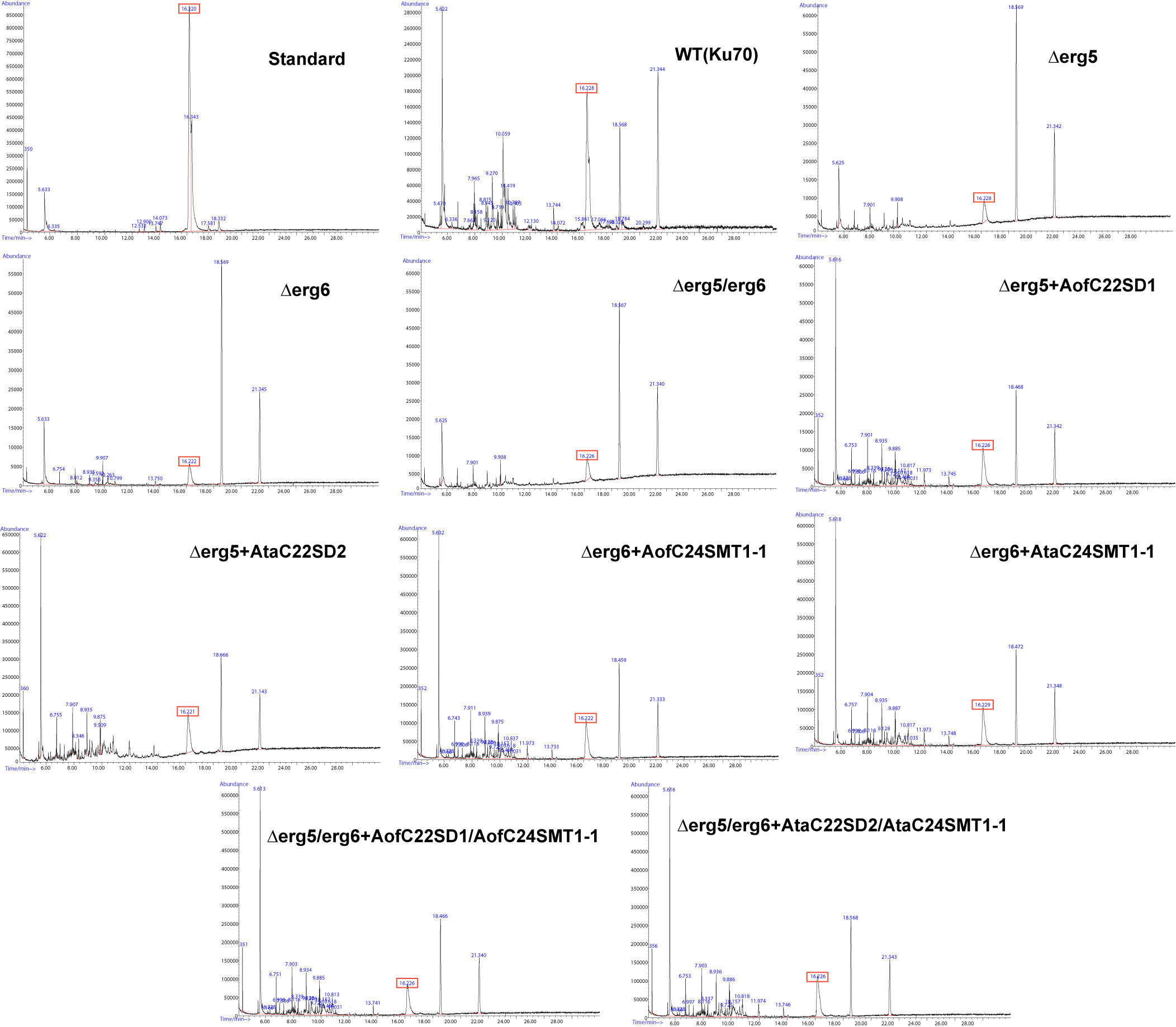

Supplement: Supplementary Figure 10 — GC–MS chromatograms validating sterol pathway disruption and complementation in N. crassa WT (Ku70), Δerg5, Δerg6, Δerg5/Δerg6, and complemented strains expressing Asparagus officinalis or A. taliensis C22SD and C24SMT genes. Complemented strains include Δerg5+AofC22SD1, Δerg5+AtaC22SD2, Δerg6+AofC24SMT1-1, Δerg6+AtaC24SMT1-1, Δerg5/Δerg6+AofC22SD1/AofC24SMT1-1, and Δerg5/Δerg6+AtaC22SD2/AtaC24SMT1-1. Diagnostic peaks corresponding to sterol products are boxed in red. One spectrum is shown per condition; all replicates yielded highly similar fragmentation patterns [file Image10.tif]
